# miR-182-3p/Myadm contribute to pulmonary artery hypertension vascular remodeling via a KLF4/p21-dependent mechanism

**DOI:** 10.7150/thno.44687

**Published:** 2020-04-25

**Authors:** Lan Sun, Peirong Lin, Ying Chen, Haoying Yu, Shuyu Ren, Jingrong Wang, Liyun Zhao, Guanhua Du

**Affiliations:** 1 Institute of Materia Medica, Chinese Academy of Medical Science and Peking Union Medical College,1 Xian Nong Tan Street, Beijing 100050, China.; 2 The State Key Laboratory of Bioactive Substance and Function of Natural Medicines 1 Xian Nong Tan Street, Beijing 100050, China; 3 Beijing Key Laboratory of Drug Targets Identification and Drug Screening Beijing 100050, China.; 4 Department of anesthesiology, Beijing Anzhen Hospital, Capital Medical University, and Beijing Institute of Heart, Lung, and Blood Vessel Diseases, Beijing 100029, China.

**Keywords:** pulmonary artery hypertension, microRNA, myeloid, vascular remodeling, p21/Cip1.

## Abstract

**Rationale:** There is a continued need for investigating the roles of microRNAs and their targets on the pathogenesis of pulmonary arterial hypertension (PAH) vascular remodeling. We recently identified the association of myeloid miR-182-3p and its new target, Myeloid-Associated Differentiation Marker (Myadm), with vascular remodeling. Here, we aimed to determine the role of miR-182-3p/Myadm on PAH vascular remodeling and the underlying molecular mechanism.

**Methods:** The miR-182-3p/Myadm expression profiles were detected in PAH patients and experimental rodent models. Loss-of-function and gain-of-function studies using gene knock-in or gene knock-out and the combinations of the proteomic technology and genome-wide ChIP-Seq were employed to determine the downstream targets of miR-182-3p/Myadm in response to monocrotaline (MCT)-induced PAH.

**Results:** The miR-182-3p/Myadm expression was altered in PAH patients and experimental rodent models. Both miR-182-3p inhibitor and overexpression of Myadm augmented the pathological progression in rats in response to MCT-induced PAH. In contrast, miR-182-3p mimic and Myadm gene knockout attenuated the changes in the hemodynamics and structure of the cardio-pulmonary system in MCT-induced PAH in rats. Myadm mediated the proliferation of pulmonary artery smooth muscle cells (PASMCs) by altering the cell cycle kinase inhibitor (p21/Cip1) expression through the transcription factor Krüppel-like factor 4 (KLF4) translocation into the cytoplasm.

**Conclusion:** Our findings indicate the prognostic and therapeutic significance of miR-182-3p in PAH and provide a new regulatory model of the myeloid-derived miR-182-3p/Myadm/KLF4/p21 axis in PAH vascular remodeling.

## Introduction

Pulmonary arterial hypertension (PAH) is a devastating and lethal cardio-pulmonary disease with an estimated prevalence of ~15 cases per million individuals 1. Currently, following the 6th WSPH update, PAH refers to hemodynamic alterations in the pulmonary circulation resulting in a pulmonary arterial pressure of ≥20 mmHg 2. In this setting, pulmonary arteries and veins undergo neointimal formation, and obliteration of distal pulmonary arteries (PAs) has been recognized as pulmonary vascular remodeling, which is believed to contribute to an increased pulmonary vascular pressure by increasing the pulmonary vascular resistance. The hyperproliferation of pulmonary artery smooth muscle cells (PASMCs) is recognized as a promising target for intervention in PAH-related vascular remodeling, and there is a need for studies investigating the molecular mechanism to target PASMCs 3,4.

It is now well recognized that miRNAs, the small non-coding RNAs that promote degradation or suppress translation of target genes, such as miR-21, miR-1, miR-204, miR-130/301, are abnormally expressed in PASMCs isolated from PAH patients and animals and are correlated with dysregulation of PASMC functions and PAH pathogenesis 5-8. Our recent research identified that the oncogenic miR-182-3p inhibits SMC proliferation and can be a new regulator of vascular remodeling 9. miR-182 was previously shown to be positively correlated with the initiation and development of a variety of tumors because of its role in the regulation of cell migration and proliferation. Overexpression of miR-182 potently inhibited SMC proliferation and migration under both basal conditions and platelet-derived growth factor-BB stimulation 10. We also found that miR-182-3p regulated vascular remodeling via its target Myeloid-Associated Differentiation Marker (Myadm). This novel myeloid-associated marker is broadly expressed in different tissues and cells 11-14 and is reduced in hyperproliferating PASMCs. Considering the role of miR-182-3p/Myadm on vascular smooth muscle cell (VSMC) proliferation and pathological progression of SMC hyperproliferation in the peripheral and pulmonary arteries, we hypothesized that myeloid-derived miR-182-3p/Myadm affect PASMC proliferation, leading to the development of vascular remodeling in PAH. Therefore, miR-182-3p/Myadm might have diagnostic and therapeutic implications as has been previously described for other miRNAs 15.

It is important to identify the underlying molecular mechanisms of miR-182-3p/Myadm and the potential downstream targets involved in the regulation of PASMC proliferation. Overwhelming evidence has implicated the transcription factor Krüppel-like factor 4 (KLF4) in the pathogenic behavior of PASMCs 16, 17. However, whether myeloid-associated genes such as miR-182-3p/Myadm have direct effects on KLF4 that contribute to the aberrant function of PASMCs remains unknown but may be worth exploring as a promising target for PAH intervention. We aimed to identify the downstream regulator of miR-182-3p/Myadm using a proteome-wide screening approach in Myadm^-/-^rats induced with experimental PAH, focusing on KLF4. The data identified p21/Cip1 as the most significantly altered protein by Myadm in response to MCT-induced PAH. Thus, our investigation established a new regulatory “miR-182-3p/Myadm/KLF4/p21” axis suggesting the therapeutic significance of miR-182-3p in PAH and the possibility that the myeloid differentiation-directed modification of genes such as Myadm is a potential therapeutic target for PAH.

## Methods

### Human plasma

Plasma samples were obtained from 46 patients with PAH defined by the 2013 Nice classification 18 and 71 control subjects, including patients with hypothyroidism, osteoporosis, nephrolithiasis, diabetes mellitus, osteoarthritis, and hypertension.

### Generation of gene knockout rats, animal models of pulmonary hypertension (PH) and hemodynamic measurements

All experiments were approved by the Animal Studies Committee of Peking Union Medical College.

Myadm^-/-^ rats were generated using the CRISPR/Cas9 system 19 at the Beijing Biocytogen Co.,Ltd. In brief, guide RNAs were designed both upstream (5ʹ- CCTGGATTCCTCAGTATACCAGA) and downstream (5ʹ- CCTGTTGGATACGTATGCTCTTC) of the rat Myadm gene. Fertilized eggs were collected from female Sprague-Dawley (SD) rats (Beijing Vital River Laboratory Animal Technology Co., Ltd.), and were administered pregnant mare serum gonadotropin and human chorionic gonadotropin to induce superovulation for pronuclear injection. The guide RNAs and Cas9 mRNA were injected into the eggs, which were transplanted into pseudo-pregnant SD females to generate founder rats. The genotypes of the founder rats were analyzed by PCR and direct sequencing of tail DNA samples to confirm gene deletion. The primer sequences were forward 5′-GCAGGGCTAGGATGAGCTTGGAATC and reverse 5ʹ-AGACAAGAATTCTGGGCTCCCAAG. Myadm^+/-^ parents were mated to generate Myadm ^+/+^, Myadm ^+/-^, and Myadm^ -/-^ rats. The rats were maintained in cages with three or fewer animals per cage with free access to chlorinated water and food. The conditions in the animal quarters were set at 23 °C ± 2 °C (allowable range: 18 °C to 28 °C), humidity 55% ± 10% (allowable range: 30% to 70%), and 12 h lighting cycle (lights on 7:00 to 19:00). The acclimation period was >3 days.

### Animal models of PH and hemodynamic Measurements

Hypoxia-induced and monocrotaline (MCT)-induced PH models of mice, wild-type rats, and Myadm gene knockout (Myadm^-/-^) rats were used in this study. In the hypoxia-induced PH model, the animals were exposed to hypoxia (10% O_2_) in a ventilated chamber for 3 weeks. Control animals were exposed to normoxic conditions. In the MCT-induced PH model, the MCT (Sigma, Saint Louis, MO, USA) was dissolved in 0.5 N HCl to 200 mg/mL, neutralized to pH 7.4 with 0.5N NaOH, and then diluted with sterile water to 60 mg/mL. One dose of MCT (60 mg/kg body weight) was injected subcutaneously into animals. Control rats were injected with the equivalent volume of dissolvent solution according to their weights. Food and water were provided ad libitum, and the animals are checked once per day. On day 21 after exposure to hypoxia or day 28 after MCT injection, rats developed PH as demonstrated by significantly increased right ventricular systolic pressure (RVSP, an average of right ventricular peak systolic pressures) and pulmonary artery systolic pressure (PASP).

After a set duration of MCT exposure, rats were weighed and anesthetized with pentasorbital sodium 30 mg/g body weight injected intraperitoneally. B-mode, M-mode, and pulmonary pulsed-wave Doppler echocardiography were performed by a Visual Sonics Vevo 2100 device (Fuji Film VisualSonics, Toronto, ON, Canada) equipped with a 30-MHz linear transducer. The pulmonary velocity maximum (PVmax) and the tricuspid annular plane systolic excursion (TAPSE) were used to assess the cardiac and pulmonary hemodynamics of PAH animals. RVSP and PASP were determined by right-sided heart catheterization with a Millar pressure transducer catheter prefilled with heparinized saline that had a 90° angle to the shaft at the distal end. When it was inserted into the right jugular vein of a rat, it straightened out and then resumed its original shape once it was located in the right ventricle. It was further advanced so that the tip slipped through the pulmonary valve and then the artery pressure was detected using BL-420S physiological experiment system. (Chengdu Techman, Sichuan, China). A weight ratio of the RV divided by the sum of left ventricle and septum was measured to determine the extent of right ventricular remodeling.

### Immunohistochemistry, immunofluorescence, and imaging

The animals were anesthetized and perfused with 0.1 M phosphate buffered saline, fixed with 4% paraformaldehyde. Whole heart, lung, and pulmonary artery samples were embedded in paraffin, sliced into 5 μm sections and then were stained for hematoxylin and eosin (H&E), immunofluorescence, wheat germ lectin (WGA) and sirius red followed by examination with a light microscope (Nikon).

Pulmonary artery remodeling was assessed with Image Pro Plus software (version 11) after lungs were stained with H&E. A minimum of 10 microscopic fields were examined for each slide. Approximately 20 muscular arteries with diameters of 50 to 100 or <50 μm per lung section were outlined and measured. Vessel remodeling was calculated as [(external vessel area-internal vessel area)/ external vessel area], as previously described 20. Occlusive pulmonary arteries were counted from H&E-stained lung slides from each group.

### Cell culture

Rat primary pulmonary artery smooth muscle cells (PASMCs) were cultured as follows. The lungs were removed and, under a dissection microscope, the third- and fourth-generation pulmonary arteries (PAs) (∼300 to 800 μm) were isolated and cleared of connective tissue. The endothelium was then removed and the PASMCs were enzymatically isolated and transiently cultured as previously described 21. The cells used in all of the experiments were between the fourth and seventh passages and were cultured on conventional uncoated dishes.

HEK293a cells were cultured in Dulbecco's Modified Eagle Medium (DMEM). The cultures were supplemented with 10% fetal bovine serum (FBS) and 100 μg/mL penicillin/streptomycin.

### Cell proliferation assays

The cell proliferation rate was measured using Cell Counting Kit-8 or using 4',6-diamidino-2-phenylindolestaining (DAPI). The DNA synthesis in proliferating cells was detected using EdU incorporation. For CCK-8 staining, two hours before detection, 10 μl CCK-8 reagent (Dojindo, Tokyo, Japan) was added, incubated, and the absorbance at 450 nm was detected using a microplate reader (Thermo Fisher Scientific, Waltham, MA USA) to assess cell proliferation. For DAPI staining, at the end point, cells were immediately fixed and permeabilized, and the DAPI labeled cells were examined using a Cellomics ArrayScan VTI HCS Reader (Thermo Fisher Scientific, Ottawa, ON, Canada). For the EdU incorporation assay, the PASMCs were seeded at 50% confluence in Costar 96-well plates. The cells were then starved in an FCS-free medium for 48 h followed by various treatments. Six hours before harvesting, EDU was added to the medium (10 μM), immediately fixed, and permeabilized. The cells were washed twice with PBS, 1 mL of 0.5% Triton X-100 in PBS was added to each well and incubated for 20 minutes at room temperature. Subsequently, EdU staining was performed using an EdU staining kit (Ribobio, Guangzhou, China) according to the manufacturer's instructions. DAPI (BIOMOL, Enzo Life Sciences, NY, USA) was employed to detect the nuclei. The labeled cells were examined using a Cellomics ArrayScan VTI HCS Reader (Thermo Scientific, Ottawa, ON, Canada).

### Plasmid construction, transient transfection and dual-luciferase assay

The luciferase reporter plasmid carrying the human CDKN1A (p21/Cip1) promoter (pGL3-p21/Cip1, nucleotides: 5 kb to 30 bp) was cloned between the Mlu I and Xho I sites in the pGL3-basic vector as previously described 22. The human KLF4 overexpression plasmid (pcDNA-KLF4) was also constructed. KLF4 cDNA was amplified by two rounds of PCR with different oligonucleotide primers mix (See Table 1). The PCR product was cloned into the plasmid pcDNA3.1 between the BamHI and EcoRI sites after the plasmid was linearized. Separately, a control plasmid (Control-pcDNA) was generated by cloning a green fluorescent protein expression sequence in the same position.

pcDNA-KLF4 or control plasmid was co-transfected with the p21/Cip1 promoter reporter plasmid or pRL-TK vector plasmid (Promega, Madison, WI, USA) into HEK293a cells using the liposome technology (FuGENE HD; Roche, Indianapolis, IN, USA) according to the manufacturer's protocol. The cells were harvested 48 h after transfection. Luciferase activity was determined using a dual-luciferase assay system (Promega, Madison, WI, USA) according to the manufacturer's instructions. Firefly luciferase activities were measured using SpectraMax M5 Microplate Reader (Molecular Devices, Sunnyvale, CA, USA) and normalized against *Renilla* luciferase activity.

### Immunofluorescence

Cell immunofluorescence analyses were performed using anti-p21(Abcam, Cambridge, MA, US), and anti-KLF4 (Proteintech, Wuhan, China) as primary antibodies, followed by FITC (green fluorescence, Chemicon, Temecula, CA, USA)-conjugated secondary antibodies and visualized by Cellomics array scan (ArrayScan VTI HCS Reader; Cellomics). The nuclei were visualized by DAPI staining.

For the tissue immunofluorescence staining, sections were incubated with anti-SM-α actin (SMA, 1:50, Santa Cruz, Texas, USA) and anti-Myadm (1:50, Novus, Littleton, CO, USA) antibodies, followed by fluorescein-conjugated secondary antibodies (1:300 dilution). The cell nuclei were stained with DAPI.

### Western blot analysis

For Western blotting, anti-β-actin, anti-CyclinD1, anti-CDK2 (Cell Signaling, Danvers, MA, USA), anti-SMAD4, anti-p21/Cip1 (Abcam, Cambridge, MA, USA), anti-KLF4 (Proteintech, Wuhan, Hubei, China), anti-proliferating cell nuclear antigen (anti-PCNA) (Proteintech, Wuhan, Hubei, China), and anti-myeloid-associated differentiation marker (anti-Myadm) (Novus, Littleton, CO, USA) antibodies were used as the primary antibodies. The signals were detected using the enhanced chemiluminescence detection method and were quantified by densitometry.

### Generation of recombinant adenovirus

The recombinant adenovirus vectors overexpressing miR-182-3p or miR-182-3p inhibitor were described previously 9. The recombinant adenovirus vectors that overexpress rat Myadm gene (Ad-Myadm) or express small interference RNA (siRNA) against the Myadm gene (Myadm-siRNA) were constructed. The short hairpin RNAs (shRNAs) with the sequences targeting Myadm mRNA and Myadm cDNA were amplified by two rounds of PCR with different oligonucleotide primer mix (See Table 2 and Table 3). Subsequently, the PCR products were cloned into the shuttle plasmid pAdTrack-CMV between the EcoRI and BamHI sites after the plasmids were linearized. The plasmids containing unrelated sequences were used as negative controls (Ad-Control and siRNA-Control, respectively). The adenoviruses expressing Myadm or si-Myadm or controls were packaged using the RAP Ad Universal Adenoviral Expression System (Cell Biolabs) from GenePharma Biotechnology Inc. (GenePharma, Shanghai, China).

### RNA isolation and real-time polymerase chain reaction (RT-PCR)

Total RNA from lungs and plasma was isolated using Trizol and the extracted RNA was reverse transcribed in the presence of a poly-A polymerase with an oligo-dT adaptor. RT-PCR was carried out with SYBR green detection with a forward primer for the mature rno-miR-182-3p sequence (which, is the same as mature hsa-miR-182-3p) and a universal adaptor reverse primer. Relative expression was evaluated by the comparative Ct (threshold cycle) method and normalized to the expression of U6 small RNA. Primers were from Gene Copoeia (hsa-miR-182-3p Mature_ACC: MIMAT0000260); hsa-RNU6 Mature_ACC:NR_002752.1).

### Proteomic profiling of MCT-induced PAH rats by iTRAQ-coupled 2-D LC/MS/MS

**Sample processing and labeling.** Lungs isolated from MCT- and hypoxia-induced PAH wild-type (WT) or Myadm-/- rats were washed with cold 0.1M PBS three times. The tissues were grinded and lysed with 200 μM lysis buffer (7M urea, 1.4 M sulfourea, 4%w/v CHAPS, and 0.05% w/v SDS) on ice at 70HZ using a homogenizer for 180 s. After 20 mins, the lysates were sonicated with an ultrasound sonicator, centrifuged at 14000×g for 30 min at 4 °C to remove debris, and the supernatants were collected. After the proteins were reduced, cysteine blocked, and digested according to iTRAQ labeling protocol supplied by the manufacturer (Applied Biosystems), each sample was labeled with the iTRAQ reagent: iTRAQ tags 113, 114, 115, and 116 were used for MCT-treated WT rats, normal saline-treated WT rats, MCT-treated Myadm^-/-^ rats and MCT-treated WT rats. The labeled samples were then pooled for further LC/MS analysis.

#### Two-dimensional separation of peptides using RP-RP-HPLC system

After iTRAQ labeling was completed, 2D-LC separation of the labeled tryptic peptides was carried out as follow: the samples were dried down and resuspended in SCX loading buffer. SCX separations were performed on a RIGOL-L3000 HPLC system; using the Agela Durashell-C18 reversed phase column (4.6×250mm, 5 μm) at a flow rate of 0.7 mL/min. Buffer A was 2% ACN-98% H2O (pH:10.0) and buffer B was 98% ACN-2% H2O (pH:10.0). The gradient was Buffer B at 5%-8% (0-5 minutes following sample injection), 8%-32% (5-35 min), 32%-95% (35-62 min), 95% Buffer B (64-68 min), 95%-5% (68-72 min) and switched back to 100% A to re-equilibrate for the next injection. The signal was detected by L-3500 UV-VIS Detector at 214 nm. The effective gradient eluants were combined into one fraction and collected. All 30 of these SCX fractions were dried down completely to reduce volume and to remove the volatile ammonium formate salts, then resuspended in 20 µl of 1.9% (v/v) methyl alcohol, 0.1% (v/v) methane acid and filtered prior to reverse phase C18 nanoflow-LC separation.

#### Online 2-D nano-liquid chromatography

Two enrichment columns were alternatively switched into the solvent path of the nano-pump by a 10-port valve. In the second dimension, separation by nanoflow LC system (Thermo Scientific EASY-nLC 1000 System) interfaced with a Thermo Q-Exactive™ mass spectrometry. Each SCX fraction was enriched by Acclaim PepMap100 column (2cm x 100μm, C18, 5 μm) and then auto-injected onto an EASY-Spray column (12 cm x 75 μm, C18, 3μm). Peptides were eluted using a solvent composed of buffer C and buffer D with a nanoflow gradient of buffer D. Buffer C was 100% ultrapure water, 0.1% methanoic acid, and Buffer D was 100% acetonitrile, 0.1% methanoic acid. The elution gradient was 4%-15% D (0-5 min), 15%-25% D (5-40 min), 25%-35% D (40-65 min), 35%-95% D (65-70 min), and 95%-4% D (70-82min) at a flow rate of 330 mL/min. Mass spectrometry conditions: data collection time: 40 min, spray voltage: 2.1 KV; ion source: EASY-Spray source; DP: 100; capillary temperature: 250℃; full MS: resolution: 70000 FWHM; full scan AGC target: 1e6; full scan max.IT: 60ms; scan range: 350-1800m/z; dd-MS2: resolution: 17500 FWHM; AGC target: 5e6; maximum IT: 70ms; intensity threshold: 5.00E+03; fragmentation methods: HCD; NCE: 29%; top N: 20.

#### Data analysis and interpretation

All raw files were analyzed using the Thermo Proteome Discoverer (1.4) software platform. Each MS/MS spectrum was searched for species of Rattus norvegicus (Rat) against the UniProt database. The searches were run using the following parameters: static modification of C carboxyamidomethylation (57.021 Da), dynamic modification of M Oxidation (15.995 Da) and N-Term K, iTRAQ8plex. Relative quantification of proteins in the case of iTRAQ was performed on the MS/MS scans and was the ratio of the areas under the peaks at 113, 114, 115, and 116 Da, which were the masses of the tags that corresponded to the iTRAQ reagents. The calculated peak area ratios were corrected for overlapping isotopic contributions and were used to estimate the relative abundances of a particular peptide. The unused protein score was ProteinPilot's measurement of protein identification confidence considering all peptide evidence for a protein, excluding any evidence that was better explained by a higher-ranking protein. Sequence coverage was calculated by dividing the number of amino acids observed by the protein amino acid length. The following criteria were required to consider a protein for further statistical analysis: two or more high confidence (>95%) unique peptides had to be identified, the p value in the Protein Quant had to be p <0.05, and the-fold difference had to be greater than 1.2. The candidate proteins were carefully examined in the Protein ID of the ProteinPilot software. The peptides without any modification of a free amine in the amino terminus or without iTRAQ modification of a free amine in the lysine were excluded from calculation of the protein ratios.

### Chromatin immunoprecipitation (ChIP) sequencing analysis

**ChIP.** ChIP assays were performed as described by “ChIP-Sequencing Guidelines and Practices” of the ENCODE and modENCODE consortia 20 with slight modifications. Briefly, PASMCs from Myadm^-/-^ rats and WT rats infected with recombinant adenovirus with or without Myadm-siRNA overexpression were collected. ChIP and DNA extraction were performed with the use of the ChIP-IT high-sensitivity kit from Active Motif. DNA-proteins were cross-linked with 1% paraformaldehyde and sheared (average 300 bp) with 10-s pulses using Cole-Parmer's GEX 130 Ultrasonic processor (Vernon Hills, IL) set to 30% of maximum power. DNA-protein complexes were immunoprecipitated with ChIP quality Abs (KLF4, catalog no. 11880-1-AP), using a Magna ChIPTM G kit (Millipore, Billerica, MA). The precipitates were washed three times, de-cross-linked, and subjected to qPCR. The samples were analyzed by quantitative reverse transcription polymerase chain reaction (qRT-PCR). The following primers were used: p21/Cip1 primers: F, 5′- AACCCTAAACATGGCATTCGC -3′, and R, 5′- GGCTCTAGCAGACCCTCAAAA -3′. The results were presented as mean ± SE normalized to input.

#### ChIP-Seq

10 ng of DNA samples were prepared for Illumina sequencing as the following steps: 1) DNA samples were blunt-ended; 2) A dA base was added to the 3' end of each strand; 3) Illumina's genomic adapters were ligated to the DNA fragments; 4) PCR amplification was performed to enrich ligated fragments; 5) Size selection of ~200-1500bp enriched product using AMPure XP beads. The completed libraries were quantified by Agilent 2100 Bioanalyzer. The libraries were denatured with 0.1 M NaOH to generate single-stranded DNA molecules, captured on Illumina flow cell, and amplified * in situ*. The libraries were then sequenced on the Illumina HiSeq 4000 following the HiSeq 3000/4000 SBS Kit (300 cycles) protocol.

#### ChIP-Seq Data Analysis

Sequence reads were generated from Illumina HiSeq 4000, image analysis and base calling were performed using Off-Line Basecaller software (OLB V1.8.0). After passing Solexa CHASTITY quality filter, the clean reads were aligned to the rat genome (RN5) using the BOWTIE software (V2.1.0). MACS v1.4 (Model-based Analysis of ChIP-seq) software was used to detect the peaks from ChIP-seq data. Statistically significant ChIP-enriched regions (peaks) were identified by comparison of IP vs input or comparison to a Poisson background model, using a p-value threshold of 10^-5^. The peaks in samples were annotated by the nearest gene (the nearest transcription start site (TSS) to the center of the peak region) using the newest UCSC RefSeq database. We divided the peaks into five classes on the basis of their distances to UCSC RefSeq genes, including promoter, upstream, intron, exon, and intergenic peaks.

### Statistical Analysis

Data were expressed as Mean ± SD and analyzed for statistical significance by the unpaired Student t-test or ANOVA that followed the normal distribution and met variance homogeneity. Significance was assessed with the Tukey criterion for pairwise mean comparisons under the ANOVA model. Normality was assessed with the Kolmogorov-Smirnov test. When homogeneity of variance was not met under the ANOVA model, a robust ANOVA was performed. When the data did not follow the normal distribution, P values were computed with nonparametric Kruskal-Wallis (Mann-Whitney) methods. Two-tailed p values <0.05 were considered significant after adjustment. Pearson Correlation coefficient was detected to identify the correlation between plasma levels of miR-182-3p and systolic pulmonary artery pressure (sPAP) in PAH patients. Computations were performed with the use of SPSS software (version 18.0).

## Results

### miR-182-3p/Myadm expression is altered in PH patients and in experimental rodent PH models

**Figure 1:**
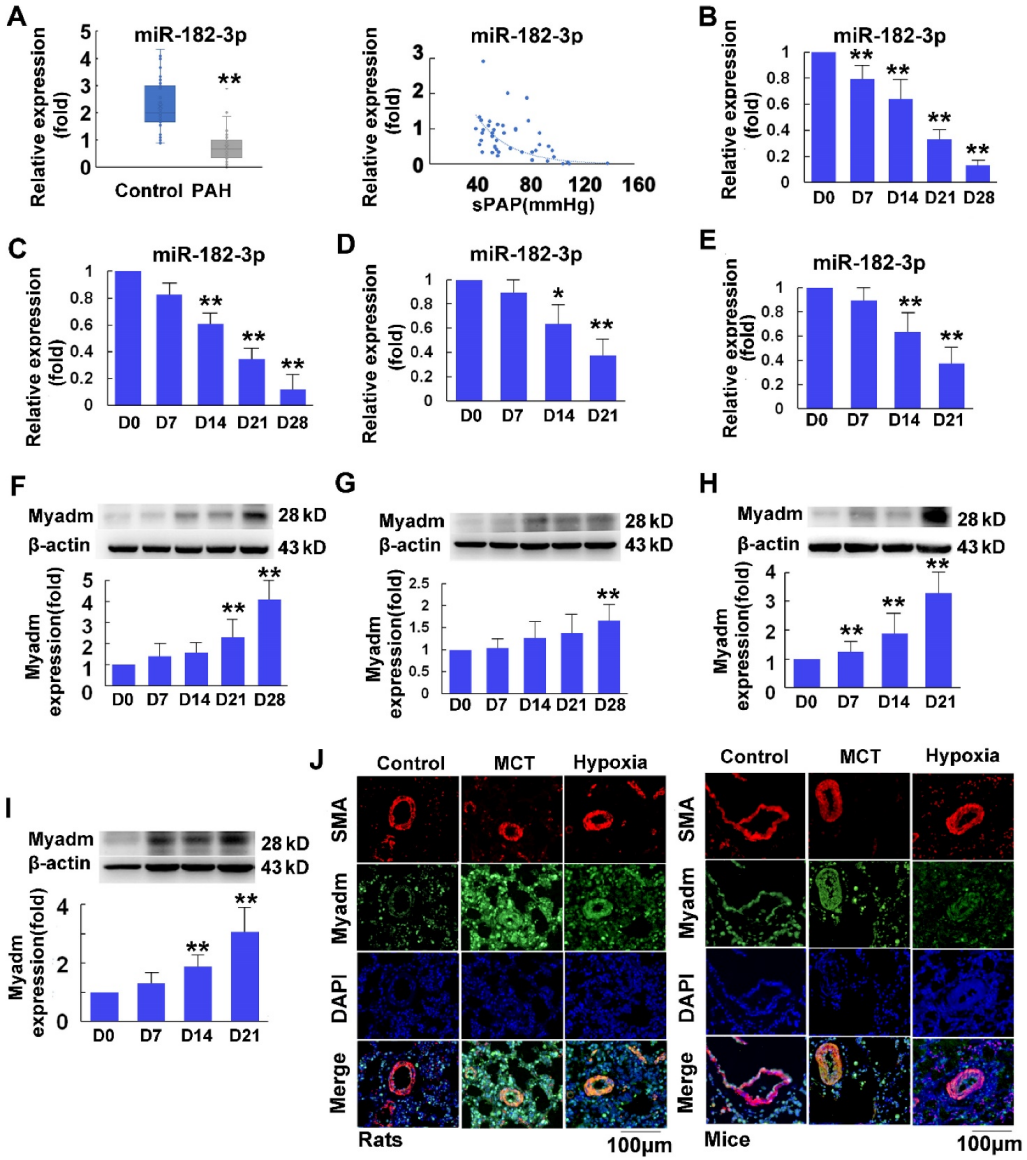
Plasma miR-182-3p levels in pulmonary hypertension patients and miR-182-3p and Myadm protein expression levels in the lungs from PAH rodent models. A, Left panel: A boxplot graph indicates that plasma miR-182-3p levels (in relation to U48) are lower in PAH patients (n=46) (grey sub-panel) compared with control subjects (blue sub-panel) without PAH (n=71). Each point represents a relative expression level of miR-182-3p from individual patients or control subjects. Right panel: Plasma miR-182-3p levels are negatively correlated with pulmonary artery systolic pressure in PAH patients. B-C: After a 4-week administration of MCT, miR-182-3p expression levels in the lungs from the rats (B) and mice (C) were detected using qRT-PCR. D-E: After a 3-week 10% hypoxia exposure, miR-182-3p expression levels in the lungs from the rats (D) and mice (E) were detected using qRT-PCR. F-I: Myadm protein expression levels normalized to β-actin in the lungs from rats (F, H) and mice (G, I) were measured using Western blotting after a 4-week administration of MCT or after a 3-week 10% hypoxia exposure. Results are expressed as mean ± SEM. J: Representative lung tissue sections with co-immunofluorescence staining of Myadm (Green) and the smooth muscle marker SM-α-Actin (SMA, red). Cell nuclei were stained with DAPI (blue). Images are at 40× magnification, bar indicates 100 μm. n=6-8. *p<0.05, **P<0.01vs the D0 group.

The plasma miR-182-3p levels were significantly decreased in PAH patients (n=46) compared with control individuals (n=71) (Figure 1A, Table 4) and were inversely correlated with pulmonary arterial systolic pressure in PH patients, as determined by a Pearson correlation model (r=-0.487, p<0.001, Figure 1A). A similar pattern was found in different rodent models of PH, including a rat and a mouse model of MCT-induced PH (4 weeks after administration of MCT (60 mg/kg body weight; Figure 1B-C), and a rat and a mouse model of hypoxia-mediated PH (10% hypoxia for 3 weeks; Figure 1D-E). As the miR-182-3p target, Myadm expression levels in lung tissues from these different rodent models of PH were also measured. As shown in Figure 1F-I, compared with control animals injected with normal saline, rats (Figure 1F, H) and mice (Figure 1G, I) injected with MCT or exposed to hypoxia appeared to exhibit increased Myadm expression levels in the lungs. Moreover, the co-immunofluorescence staining with the smooth muscle marker SMA (red color) and Myadm (green) showed that Myadm was expressed throughout the lung (including the parenchyma and stroma). The upregulation of green fluorescence in the lung indicated that both hypoxia- and MCT-induced PAH in rodents were associated with the up-regulation of Myadm (Figure 1J). Collectively, these results suggest that miR-182-3p/Myadm may play a role in the development of PAH.

### miR-182-3p regulates pathological progression in rats in response to MCT-induced PAH

**Figure 2:**
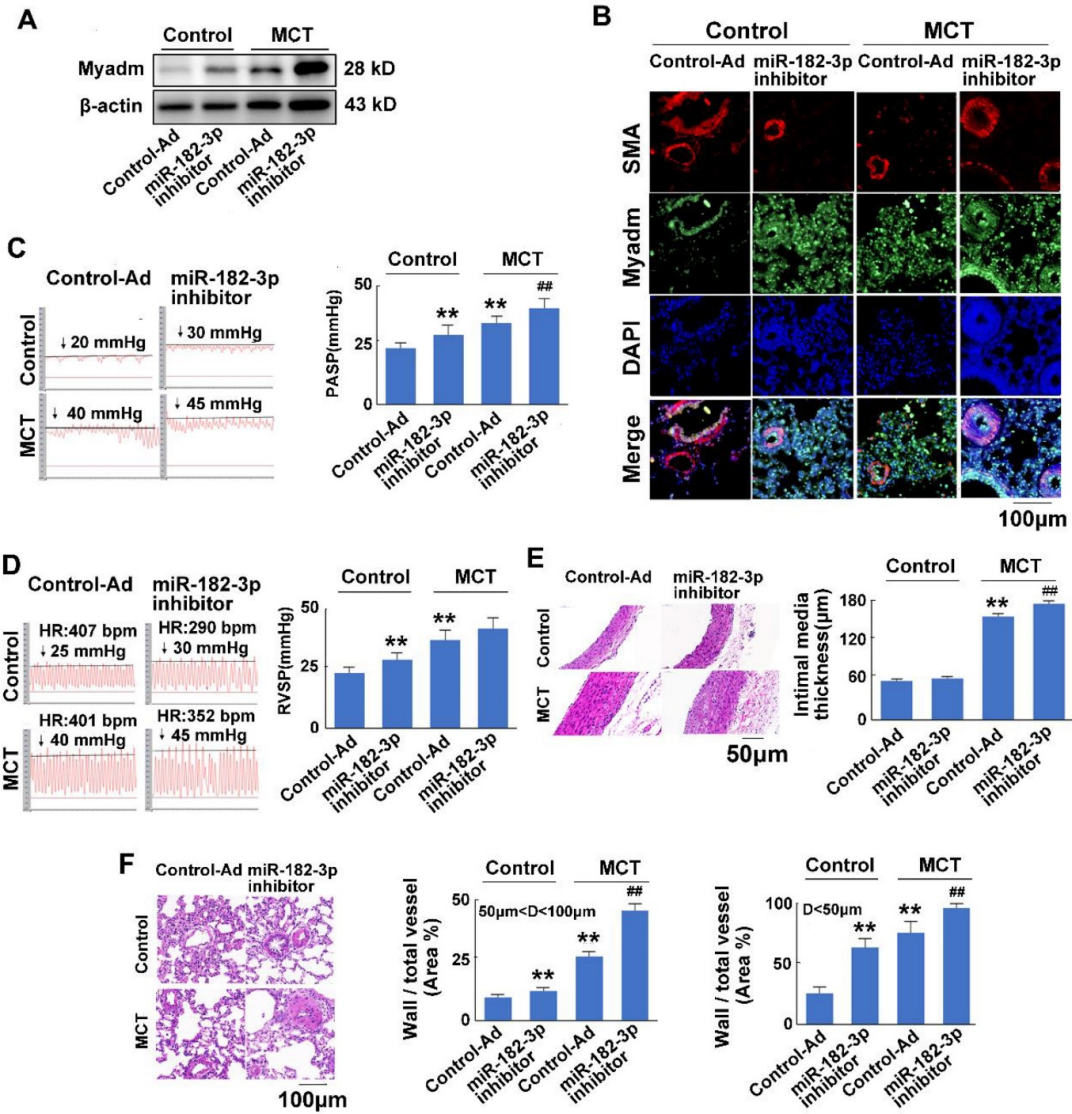
miR-182-3p inhibitor exacerbates the pathological changes in rats in response to MCT-induced PAH. Rats were transduced with recombinant adenovirus expressing inhibitor (shRNA) against the miR-182-3p (miR-182-3p inhibitor ,10^10^ pfu/mL) or the control adenovirus and then were then injected subcutaneously using one dose of monocrotaline (MCT, 60 mg/kg body weight, normal saline was used as normal control). A: The Myadm protein expression levels normalized to the β-actin in the lungs from the rats were detected using Western blotting. B: Representative lung tissue sections with the co-immunofluorescent staining of Myadm (Green) and the SMA (red). Cell nuclei were stained with DAPI (blue). Images are shown at 40× magnification. The bar indicates 100 μm. Pulmonary artery systolic pressure (PASP, C) and right ventricular systolic pressure (RVSP, D) were measured by right-sided heart catheterization with a Millar pressure transducer catheter. Black line and black arrow were used to mark the pressure values. HR: heart rates; bpm: beat per minute. Lungs and pulmonary artery samples were embedded in paraffin, sliced into 5-μm sections and stained with haematoxylin and eosin (H&E). The intimal-medial thickness (E) and the ratio of the wall area to the total vessel area of pulmonary arteries with diameters of <50 μm and 50 to 100 μm (F) were measured. Images are shown at 40× (E) and 20× (F) magnification, respectively. Bar indicates 50 μm and 100 μm. The results are expressed as the means±SEMs. *p<0.05, **p<0.01 vs rats injected with control virus. #p<0.05, ##<0.01 vs rats treated with control virus and MCT. n=6-8 per group.

To examine the effect of miR-182-3p on the development of PAH, rat models of MCT-induced PAH were used. Rats were transduced with either recombinant adenovirus expressing inhibitor (shRNA) against the miR-182-3p (Ad-miR-182-3p-inhitor ,10^10^ pfu/mL) or the mimic of miR-182-3p (Ad-miR-182-3p, 10^10^ pfu/mL). Compared with the control virus-treated rats, those infected with Ad-miR-182-3p-inhibitor or injected with MCT exhibited increased Myadm expression (Figure 2A-B), an increased pulmonary hypertension pulmonary systolic blood pressure (PASP, Figure 2C), increased right ventricular systolic pressure (RVSP, Figure 2D), decreased tricuspid annular displacement (TAPSE, p=0.07, Figure S1A), and decreased maximum pulmonary velocity (PVmax, Figure S1B). Treatment with Ad-miR-182-3p-inhibitor and MCT caused the pulmonary vascular and cardiac remodeling, which are manifested as a greater right ventricular hypertrophy (right ventricle/left ventricle + septum [RV/(LV+S)]), (Figure S1C), an increased intimal-medial thickness (IMT) of the pulmonary artery (Figure 2E), and a higher ratio of the wall area to the vessel area in pulmonary arteries with diameters of <50 μm and 50 to 100 μm (Figure 2F).

In response to the treatment with MCT, the overexpression of miR-182-3p-inhibitor in rats led to a substantial increase in the Myadm expression level in the lung tissue (Figure 2A-B) and resulted in even more unfavorable cardiac and pulmonary hemodynamics than was observed in rats not treated with miR-182-3p inhibitor, namely, higher PASP (Figure 2C), higher RVSP, (Figure 2D), lower TAPSE (Figure S1A), lower PVmax (Figure S1B), greater right ventricular hypertrophy (Figure S1C), higher IMT of the pulmonary artery (Figure 2D), a higher ratio of the wall area to the vessel area in pulmonary arteries (Figure 2E), a disorder of collagen deposition (Figure S1D), and formation of myocardial fibers (Figure S1E).

**Figure 3:**
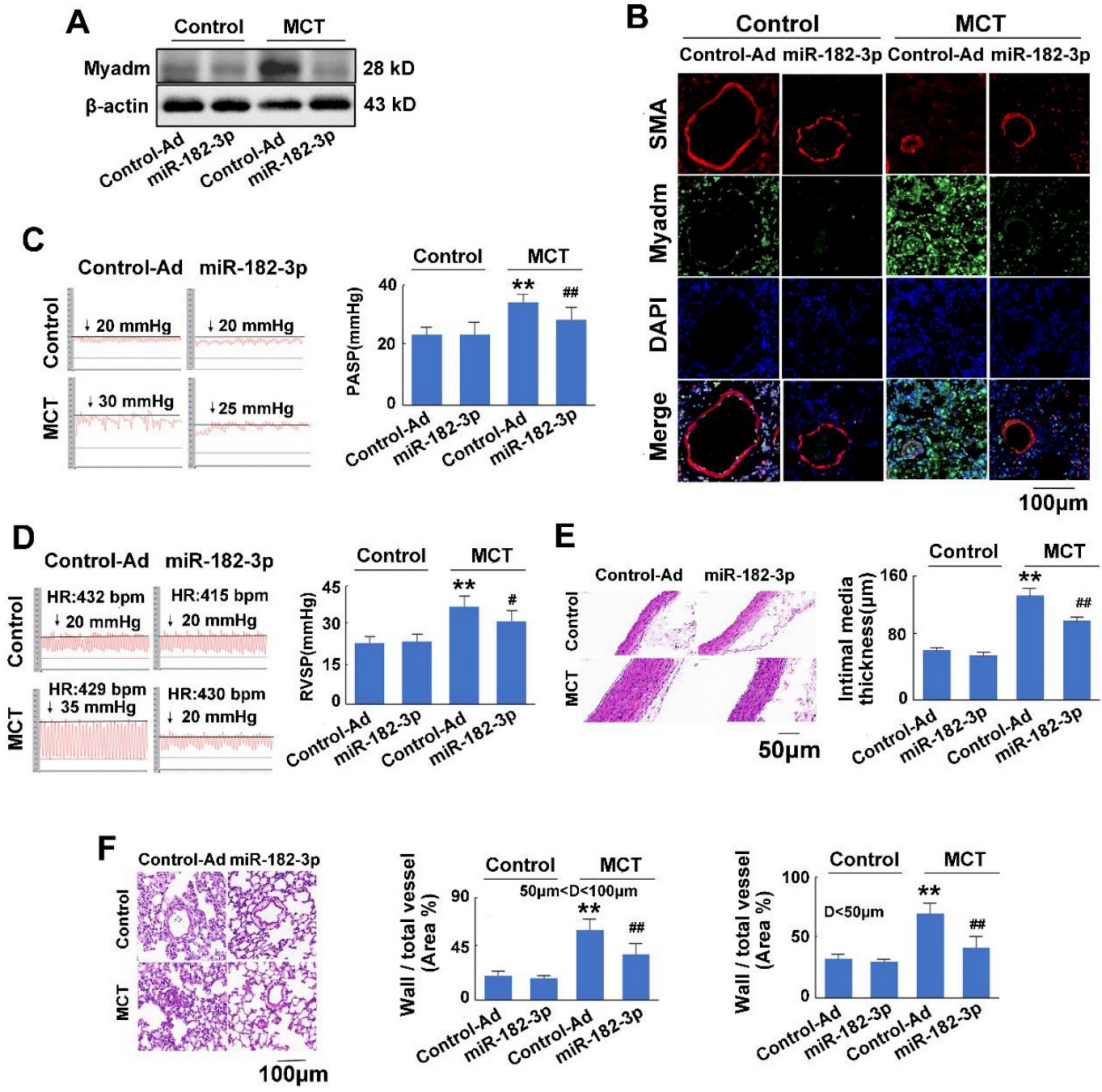
miR-182-3p regulates the pathological changes in rats in response to MCT-induced PAH. Rats were transduced with either recombinant adenovirus expressing the mimic of miR-182-3p (miR-182-3p, 10^10^ pfu/mL) or the control virus (Control-Ad) and then were injected subcutaneously using one dose of MCT. A: The Myadm protein expression levels normalized to the β-actin in the lungs from the rats were detected using Western blotting. B: Representative lung tissue sections with the co-immunofluorescent staining of Myadm (Green) and the SMA (red). Cell nuclei were stained with DAPI (blue). Images are shown at 40× magnification. The bar indicates 100 μm. PASP (C) and RVSP (D) were measured. Black line and black arrow were used to mark the pressure values. HR: heart rates; bpm: beat per minute. Lungs and pulmonary artery samples were embedded in paraffin, sliced into 5-μm sections and stained with haematoxylin and eosin (H&E). The intimal-medial thickness (E) and the ratio of the wall area to the total vessel area of pulmonary arteries with diameters of <50 μm and 50 to 100 μm (F) were measured. Images are shown at 40× (E) and 20× (F) magnification, respectively. Bar indicates 50 μm and 100 μm. The results are expressed as the means±SEMs. *p<0.05, **p<0.01 vs rats injected with control virus. #p<0.05, ##<0.01 vs rats treated with control virus and MCT. n=6-8 per group.

There was no significant difference between the rats injected with control virus and those injected with miR-182-3p mimic virus when the rats were not treated with MCT. In response to MCT, as expected, compared with the control treatment, the overexpression of miR-182-3p in rats exhibited appreciably reduced expression levels of Myadm in the lung tissue (Figure 3A-B). It also attenuated the changes in the hemodynamics and structure of the cardio-pulmonary system in the development of MCT-induced PAH in rats, as evidenced by the significant reversal of the PASP, RVSP, and PVmax values and the pulmonary vascular and cardiac remodeling (Figure 3C-F Figure S2). These results suggested that the decrease of miR-182-3p was associated with PAH vascular remodeling-related pathological changes, whereas the administration of miR-182-3p mimic regulated pathological progression in rats in response to MCT-induced PAH.

### miR-182-3p target Myadm is involved in the pathological progression of MCT-induced PAH in rats

**Figure 4:**
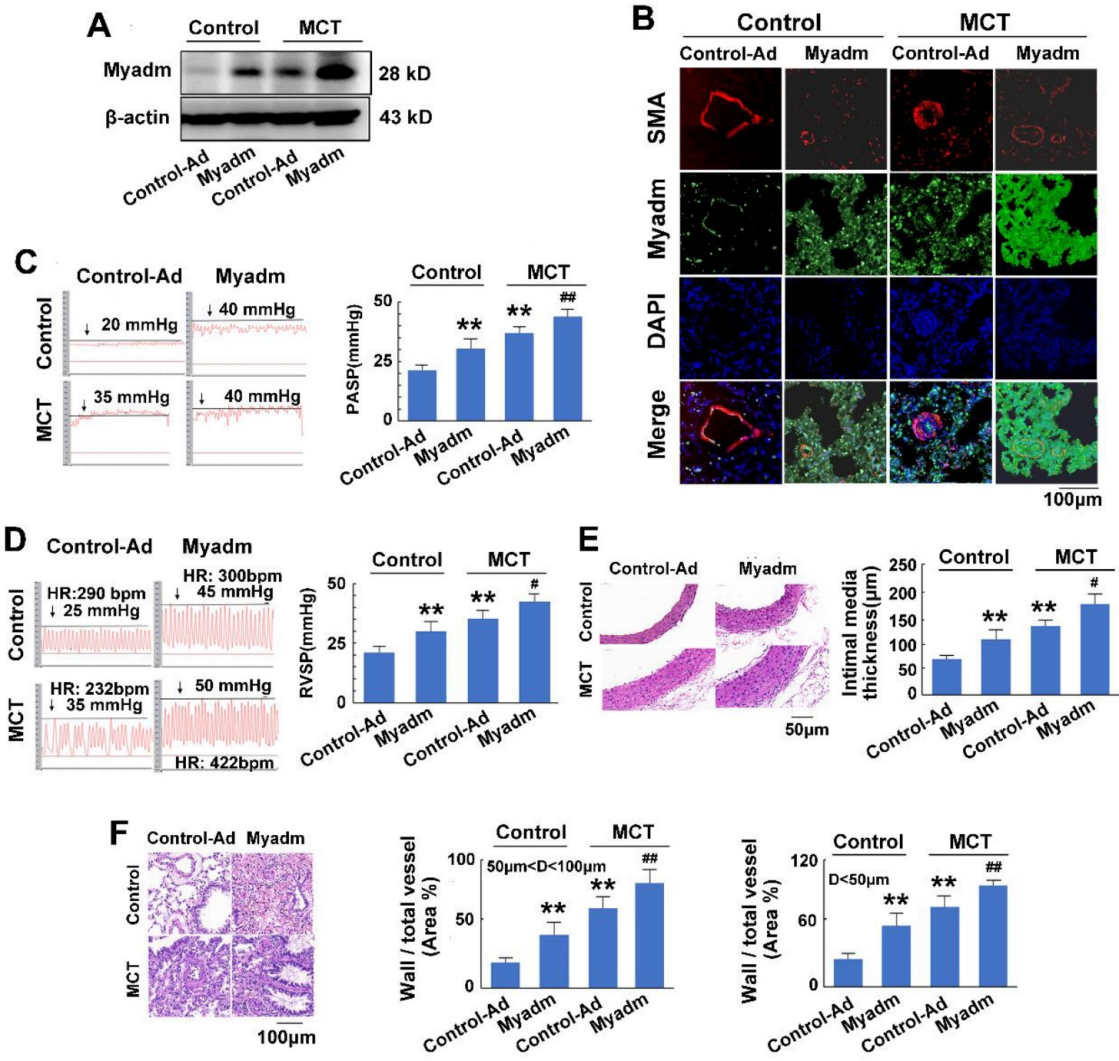
Myadm aggregates MCT-induced PAH vascular remodeling in rats. Rats were transduced with either recombinant adenovirus overexpressing the Myadm (miR-182-3p, 10^10^ pfu/mL) or the control virus (Control-Ad) and then were injected subcutaneously using one dose of MCT. A: The Myadm protein expression levels were detected using Western blotting. B: The * in situ* expression levels of the Myadm protein were detected using the co-immunofluorescent staining of Myadm (Green) and the SMA (red). Cell nuclei were stained with DAPI (blue). Images are shown at 40× magnification. The bar indicates 100 μm. PASP (C) and RVSP (D) were measured. Black line and black arrow were used to mark the pressure values. HR: heart rates; bpm: beat per minute. The intimal-medial thickness (E) and the ratio of the wall area to the total vessel area of pulmonary arteries with diameters of <50 μm and 50 to 100 μm (F) were measured after the sections were stained with H&E. Images are shown at 40× (E) and 20× (F) magnification, respectively. Bar indicates 50 μm and 100 μm. The results are expressed as the means±SEMs. *p<0.05, **p<0.01 vs rats injected with control virus. #p<0.05, ##<0.01 vs rats treated with control virus and MCT. n=6-8 per group.

We then examined the effect of the miR-182-3p target Myadm on the development of PAH. The loss-of-function and gain-of-function of Myadm were generated in rats using the CRISPR/Cas9 system (Figure S3.) or via infection with the recombinant adenovirus vectors that overexpressed the rat Myadm gene (Ad-Myadm). Compared with the controls, both Ad-Myadm-infected and MCT-treated rats exhibited higher expression levels of Myadm in the lung (Figure 4A-B), as detected by Western blotting and immunofluorescent staining. Additionally, cardiac and pulmonary changes were observed with respect to hemodynamics (increased PASP (Figure 4C), increased RVSP (Figure 4D), decreased TAPSE (Figure S4A) and decreased PVmax (Figure S4B)) structure (increased right ventricular hypertrophy (Figure S4C), increased intimal-medial of the pulmonary artery (Figure 4E), and increased ratio of the wall area to the vessel area in pulmonary arteries with diameters of <50 μm and 50 to 100 μm (Figure 4F)).

**Figure 5:**
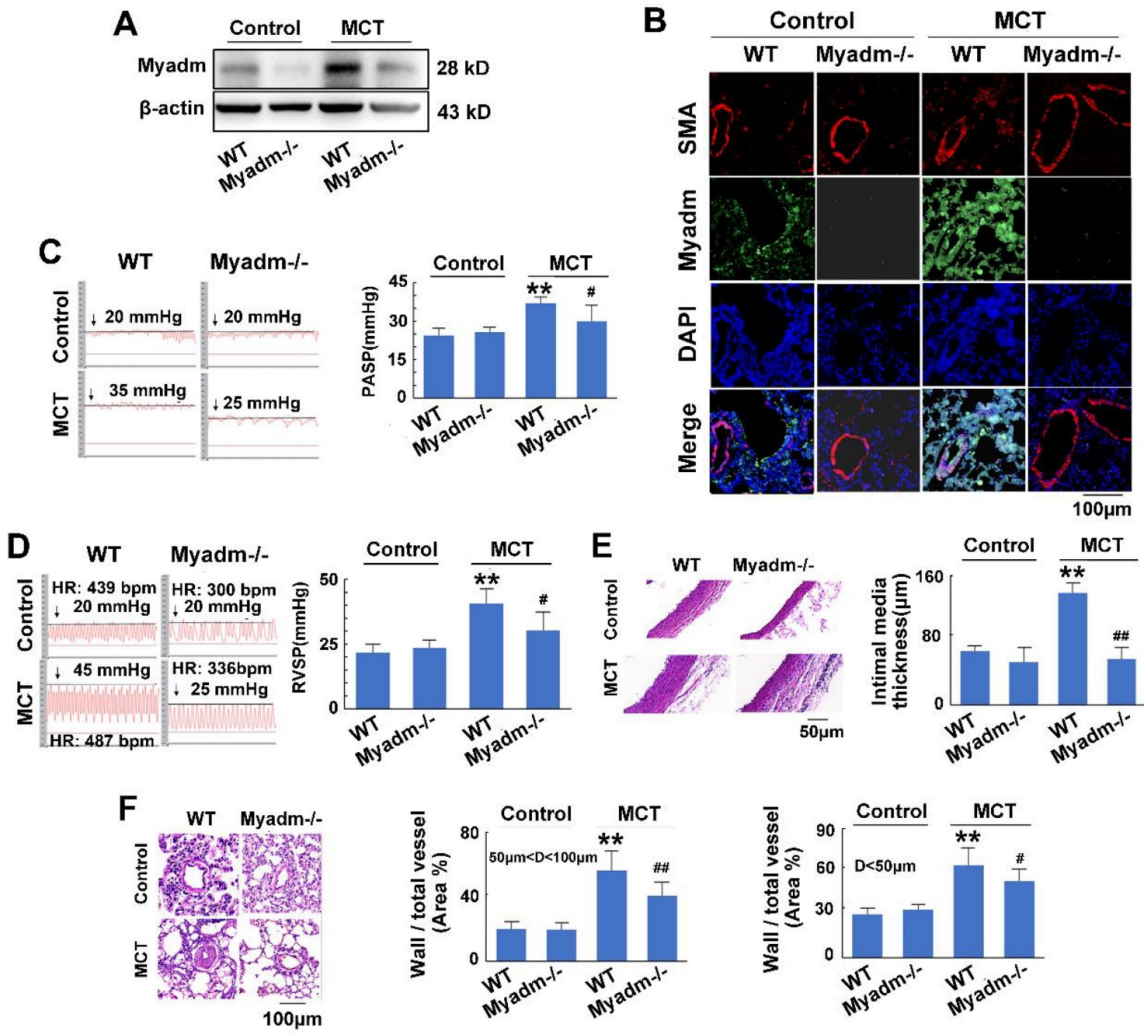
Knockout of Myadm inhibits the vascular remodelling in MCT-induced rats. Myadm gene knock out rats (Myadm^-/-^) or the wild type rats (WT) were injected subcutaneously using one dose of MCT (normal saline was used as normal control). A: The Myadm protein expression levels were detected using Western blotting. B: The * in situ* expression levels of the Myadm protein were detected using the co-immunofluorescent staining of Myadm (Green) and the SMA (red). Cell nuclei were stained with DAPI (blue). Images are shown at 40× magnification. The bar indicates 100 μm. PASP (C) and RVSP (D) were measured. Black line and black arrow were used to mark the pressure values. HR: heart rates; bpm: beat per minute. The intimal-medial thickness (E) and the ratio of the wall area to the total vessel area of pulmonary arteries with diameters of <50 μm and 50 to 100 μm (F) were measured after the sections were stained with H&E. Images are shown at 40× (E) and 20× (F) magnification, respectively. Bar indicates 50 μm and 100 μm. Results are expressed as the means±SEMs. *p<0.05, **p<0.01 vs wild type rats. #p<0.05, ##<0.01 vs wild type rats that treated with MCT. n=6-8 per group.

Furthermore, the overexpression of Myadm in rats augmented the effects of MCT, as evidenced by a variety of abnormalities. These include serious cardiac and pulmonary hemodynamics including high PASP and RVSP (Figure 4C-D), low TAPSE and decreased PVmax (Figure S4A-B), enhanced right ventricular hypertrophy (Figure S4C), increased intimal media thickness of the pulmonary artery, and high ratios of wall to vessel area in pulmonary arteries <50μm and 50 to 100μm (Figure 4E-F), collagen deposition (Figure S4D), and cardiomyocyte hypertrophy (Figure S4E). However, compared with the wild-type controls, Myadm^-/-^ rats exhibited significantly lower baseline levels of Myadm in lung tissue homogenates (Figure 5A-B) and marked protection against the pathobiology of PAH; this was evidenced by lower RVSP and PASP, decreased RVSP (Figure 5C-D), increased TAPSE and PVmax (Figure S5A-B), less severe pulmonary vascular remodeling (Figure 5E-F) and remissive fibrosis of the myocardium in response to MCT (Figure S5C-E).

In summary, the overexpression of Myadm induced PAH vascular remodeling, whereas knockout of Myadm reversed MCT-induced PAH vascular remodeling in rats. These results indicate that the effect of miR-182-3p/Myadm on vascular remodeling might be a contributor to the development of PAH.

### Myadm-mediated p21/Cip1 altered expression contributes to MCT-induced PAH in rats

**Figure 6:**
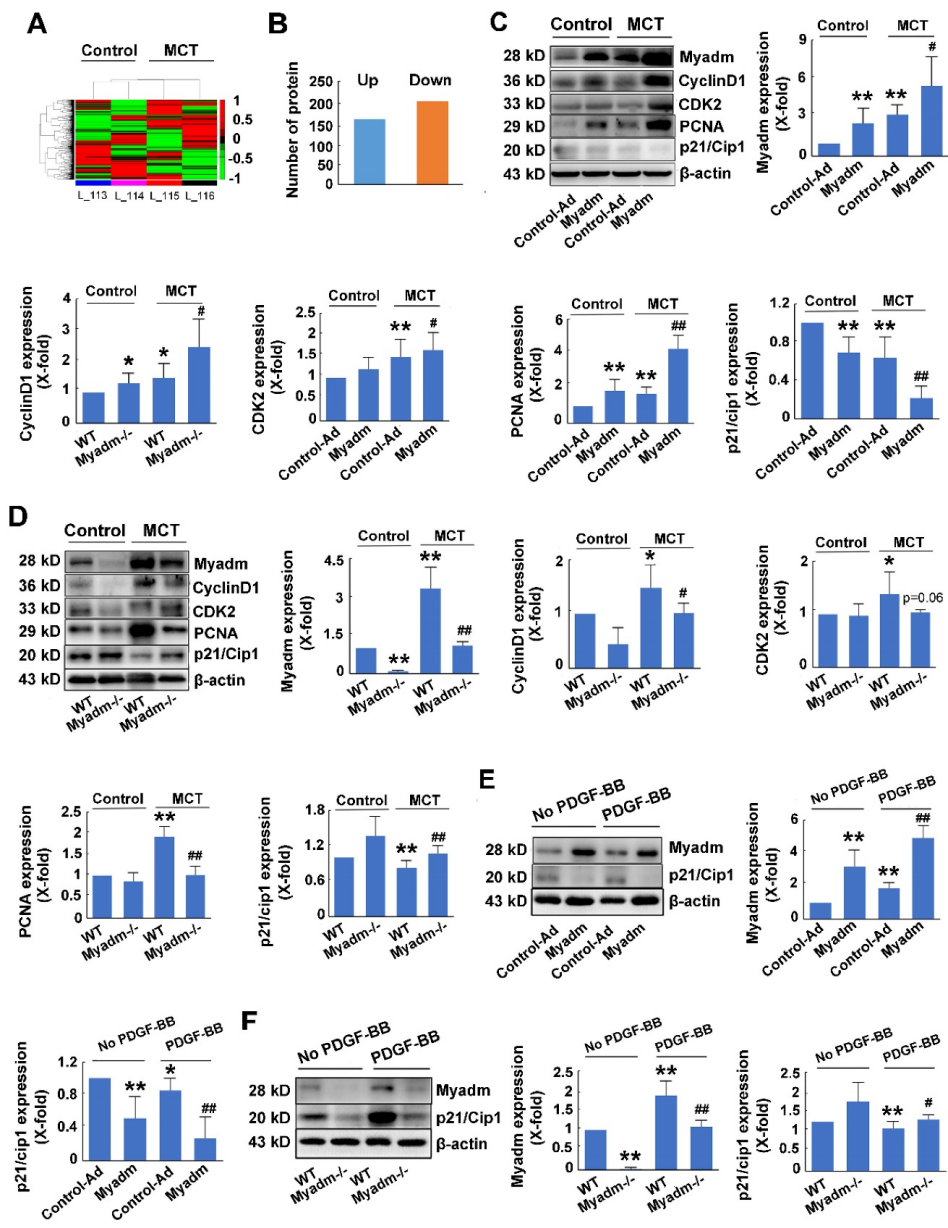
Changes in Myadm-mediated p21/Cip1 expression contribute to MCT- induced PAH in rats. Myadm-mediated proteome expression profile changes in MCT-induced PAH. A. Heatmap displaying a subset of proteins differentially expressed upon MCT-treatment in wild-types and Myadm^-/-^ rats (FC > 1.6, p <0.05), as measured by iTRAQ; B. Differentially expressed proteins in the lungs of Myadm^-/-^ rats compared to the controls, as identified by iTRAQ. n=3. C-D. Rats infected with the recombinant adenovirus overexpressing the Myadm gene (Ad-Myadm) or Myadm^-/-^ rats were injected subcutaneously using one dose of MCT, the protein expression levels of Myadm, CyclinD1, CDK2, PCNA, and p21/Cip1 normalized to β-actin in the lungs of the rats were detected using Western blotting. n=6-8. *p<0.05, **p<0.01 vs rats injected with control virus or wild-type siblings of Myadm knockout rats. #p<0.05, ##<0.01 vs rats treated with control virus/wild-type and MCT. n=6-8 per group. E-F: Quiescent rat PASMCs that overexpressed the Myadm gene (Ad-Myadm, E) or were isolated from Myadm^-/-^ rats (F) were then treated with PDGF-BB (20 ng/mL) for the next 48 hours, and the p21/Cip1 expression level was measured using Western blotting. n=6 *p<0.05, **p<0.01 vs PASMCs infected with control virus or PASMCs isolated from wild-type siblings of Myadm knockout rats. #p<0.05, ##<0.01 vs PASMCs infected with control virus or PASMCs isolated from wild-type rats in response to 10% FBS or PDGF-BB at a concentration of 20 ng/mL. n=6-8 per group.

To further delineate the cellular and molecular mechanisms underlying the regulation of PAH, a proteome-wide screening was performed using isobaric tags for relative and absolute quantitation-coupled 2D LC-MS/MS (liquid chromatography mass spectrometry) approach to identify the changes in Myadm-mediated gene expression in response to MCT-induced PAH. As is displayed in Figure 6A, compared with the wild type rats, Myadm gene knockout rats showed altered expression (P<0.05) of 227 proteins after treatment with MCT (Figure 6B), of which the p21/Cip1 was most reduced with the smallest p value (Table S1).

We then evaluated the role of Myadm in the regulation of the expression levels of p21/Cip1 in MCT-induced PAH rats. Figure 6C shows that rats overexpressing the Myadm gene or treated with MCT exhibited low expression levels of p21/Cip1 in the lung tissues, which were reduced to even lower levels in rats overexpressing the Myadm gene and were also treated with MCT. Additionally, the expression levels of cell cycle-related proteins, including PCNA, CDK2, and CyclinD1, were significantly increased in rats that overexpressed the Myadm gene. As expected, the expression level of p21/Cip1 was increased to a normal level with a reduction in the expression levels of PCNA, CDK2, and CyclinD1 in Myadm^-/-^ rats (Figure 6D).

The expression level of p21/Cip1 was also detected * in vitro* and was found to be significantly reduced in PDGF-BB-treated PASMCs (Figure 6E-F). When the PASMCs overexpressed Myadm gene, the expression level of p21/Cip1 in the PDGF-BB-treated PASMCs was further reduced to a much lower level (Figure 6E). As expected, Myadm^-/-^ PASMCs reversed p21/Cip1 expression levels to normal in response PDGF-BB (Figure 6F). These results indicate that Myadm-mediated changes in p21/Cip1 expression might contribute to MCT-induced PAH in rats.

### Myadm promotes PASMC proliferation by inhibiting p21/Cip1

**Figure 7:**
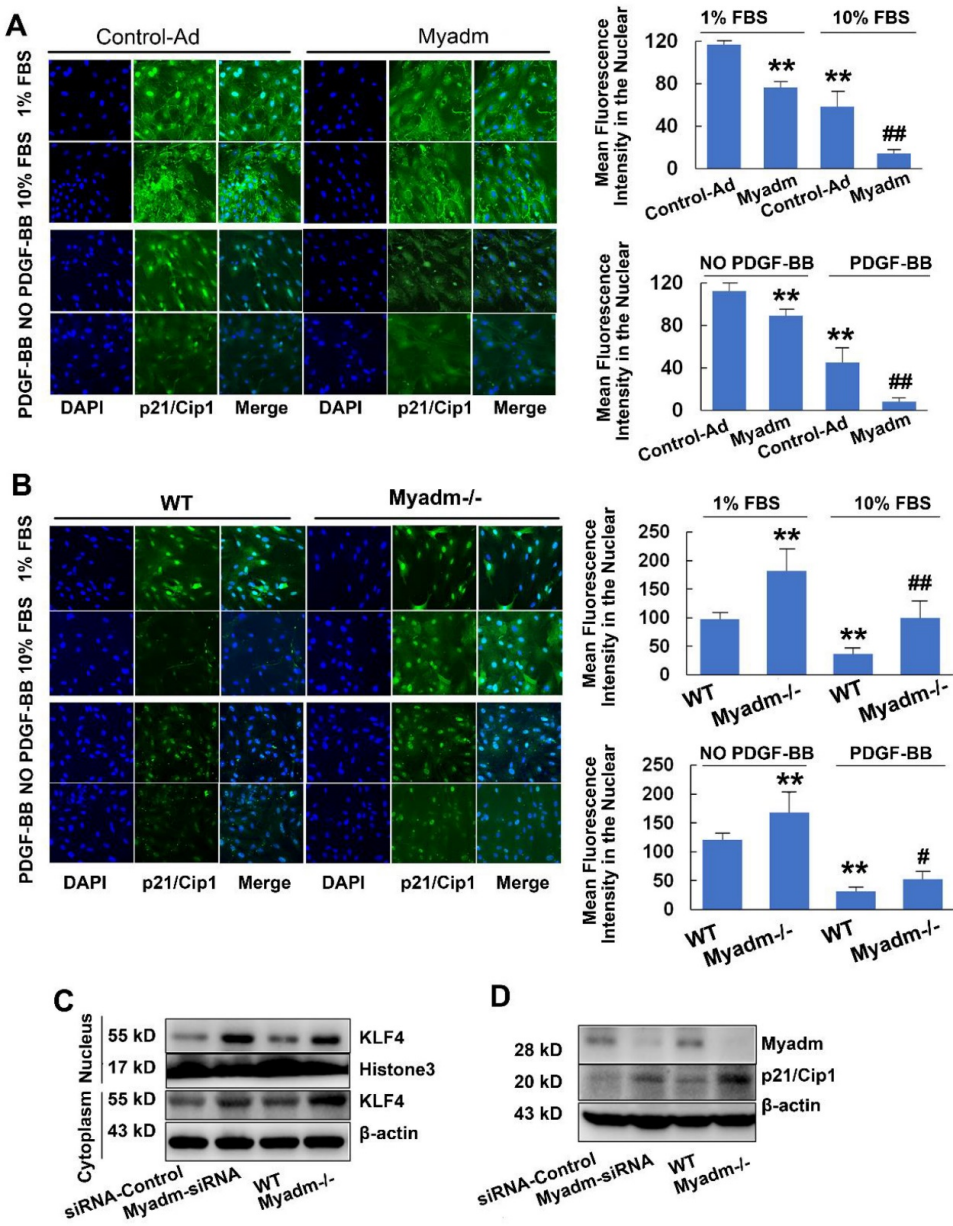
Myadm promotes PASMC proliferation by inhibiting p21/Cip1. A-B. Quiescent rat PASMCs that overexpressed the Myadm gene (Ad-Myadm) or isolated from Myadm^-/-^ rats were treated with PDGF-BB (20 ng/mL) or 10% FBS for 48 h, p21/Cip1 expression level in the nuclei was determined by immunocytofluorescence analysis. The expression level was measured by the average intensity of green fluorescence. Anti-p21/Cip1 antibodies were used as primary antibodies, followed by FITC (green)-conjugated secondary antibody and visualized by Cellomics array scan. Nuclei were visualized by DAPI staining. Images are shown at 20× magnification. Data are expressed as the fold induction compared to control. *p<0.05, **p<0.01 vs PASMCs infected with control virus or PASMC isolated from wild-type siblings of Myadm knockout rats. #p<0.05, ##<0.01 vs PASMCs infected with control virus or PASMCs isolated from wild-type rats and in response to 10% FBS or PDGF-BB at a concentration of 20 ng/mL. n=6-8 per group. C-D. Both PASMCs isolated from Myadm^-/-^ rats and PASMCs transfected with recombinant adenovirus that overexpressed small interference RNA (siRNA) against the Myadm gene were collected. Expression levels of KLF4 (both in the cytoplasm and nuclei) and p21/Cip1 were analyzed.

Since Myadm altered the expression of p21/Cip1, which is an important antiproliferative molecule, we examined whether it impacted the proliferation of PASMCs, the key to PAH-associated vascular remodeling. In either basic culture medium or medium with 10% FBS with platelet-derived growth factor-BB (PDGF-BB) , compared with the control cells, PASMCs that overexpressed Myadm (infected with recombinant adenovirus overexpressing the Myadm gene) showed markedly increased proliferation, as demonstrated by EdU incorporation and cell counting (Figure S6A-B). The p21/Cip1 expression level in the nucleus was decreased, as detected by immunocytochemistry in response to FBS and PDGF-BB (Figure 7A). As expected, the FBS and PDGF-BB-mediated proliferative capacity of PASMCs isolated from Myadm^-/-^ was reduced, as detected by the growth curve (Figure S6C),whereas the EdU incorporation rate (Figure S6D) and the expression level of p21/Cip1 (Figure 7B)were increased.

### Myadm is involved in p21/Cip1 protein expression via inhibitingKLF4 nuclear export

The translocation of KLF4 to the cytoplasm in VSMCs has been shown to regulate gene expression, including p21/Cip1, which affected a diverse array of cellular processes. Based upon the effect of Myadm on PASMC proliferation via the inhibition of p21/Cip1, we next attempted to determine the role of Myadm in the nuclear export of KLF4 detected by immunocytochemistry. As is shown in Figure S7A, the expression level of KLF4 in the cytoplasm was higher in Myadm-overexpressing cells than in control cells. Myadm overexpression significantly augmented the FBS- and PDGF-BB- induced KLF4 nuclear export, whereas the Myadm^-/-^ PASMCs completely reversed the KLF4 nuclear export in response to both FBS and PDGF-BB (Figure S7B). Also, the knockdown or knockout of Myadm in PASMCs led to an increase in KLF4 expression in the nucleus and a decrease in KLF4 expression in cytoplasm (Figure 7C), followed by a significant increase in the expression level of p21/Cip1 (Figure 7D).

**Figure 8:**
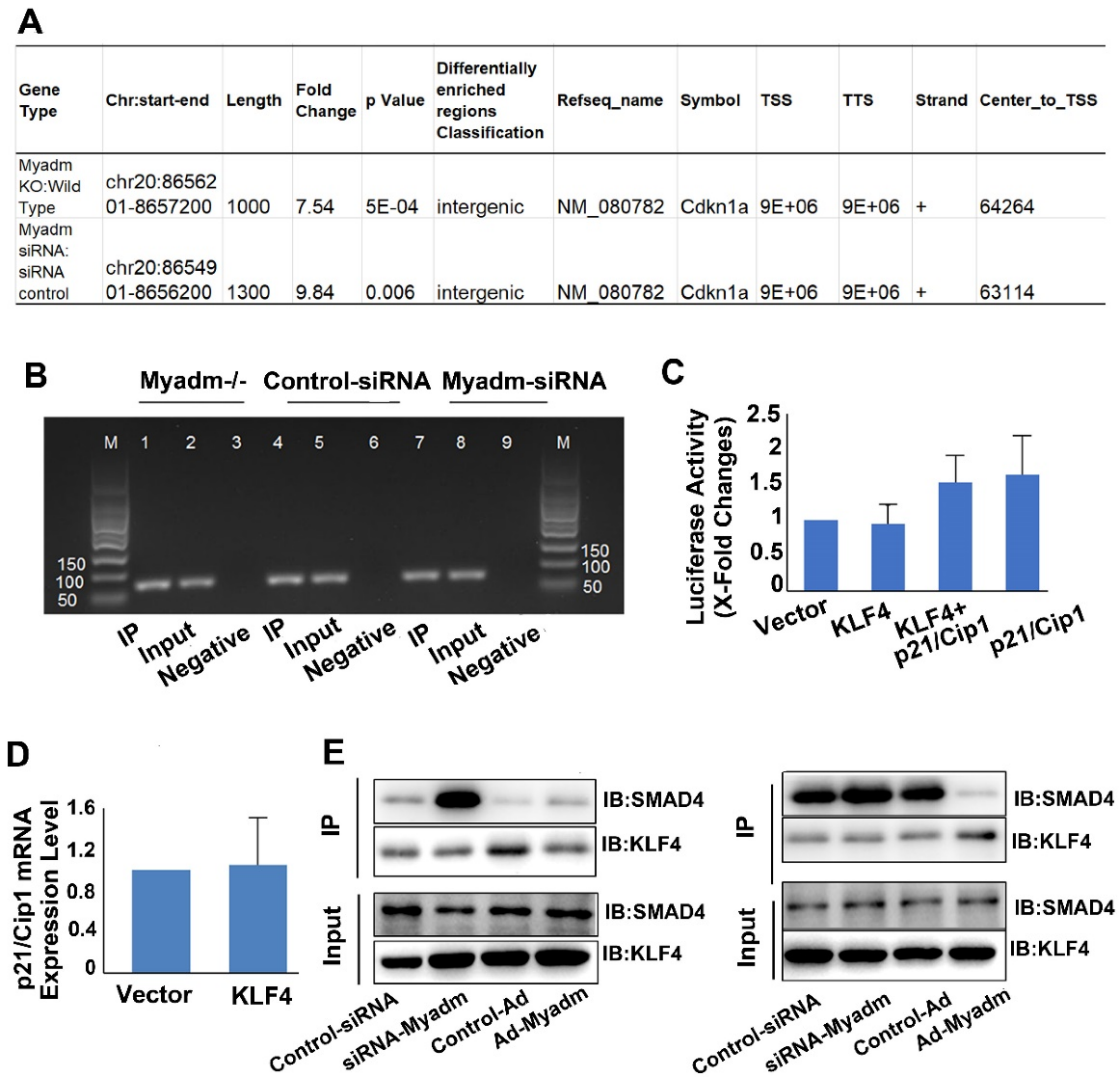
Myadm knockdown/knockout increases the binding of KLF4 to the intergenic region of the p21/Cip1 promoter and the protein expression level without changing p21/Cip1 promoter activity. PASMCs isolated from Myadm^-/-^ rats or PASMCs transfected with siRNA against the Myadm gene were used for ChIP analysis together with ChIP-seq to map the genome-wide location of KLF4 binding region followed by Myadm knockdown or knockout, focusing on the p21/Cip2 promotor. A. Myadm knockdown/knockout increased KLF4 binding to the intergenic region of p21/Cip1 promoter. B, DNA-protein complexes were immunoprecipitated with anti-KLF4 Abs and were subjected to analysis using qRT-PCR followed by agarose gel electrophoresis showing PCR product. C, Growth-arrested PASMCs were co-transfected with KLF4-overexpressing plasmid (or control) and the firefly luciferase reporter gene containing p21 promoters and the transcription activities of p21 were measured according to the luciferase activity. D, KLF4 overexpression in PASMCs did not increase the mRNA expression level of p21/Cip1. E, PASMCs transfected with Ad-Myadm or Myamd-siRNA were subjected to cross co-immunoprecipitation (IP) and Western blotting (IB) to detect KLF4-SMAD4 complex formation. In the left panel, cell lysates were immunoprecipitated with anti-KLF4 resin and then immunoblotted with KLF4 and SMAD4 antibodies. In the right panel, cell lysates were immunoprecipitated with anti-SMAD4 resin and then immunoblotted with SMAD4 and KLF4 antibodies. For C-D, n=6-8 in each group.

We then employed the ChIP technology combined with the next-generation sequencing (ChIP-seq) to map the genome-wide locations of the KLF4 binding regions followed by Myadm knockdown/knockout, focusing on the p21/Cip2 promotor. The antibody against KLF4 was used in immunoprecipitation experiments and the PASMCs isolated from WT rats served as a negative control as well as a baseline for the genome-wide expression profile. All ChIP-seq data aligned well with the reference genome. We analyzed the KLF4-enriched binding regions in the proximal promoter (NDG analysis) within 5 kb upstream or at the 5′ un-transcriptional region (UTR) from the TSS, exon, and intron 23. This analysis was based on the location of the central point of the peak region. In cases where the central point falls out of a known gene even though the peak itself overlaps the gene, the overlapping regions are defined as “intergenic” regions 24. As shown in Figure 8A, Myadm knockdown led a significant increase in KLF4 binding to the intergenic regions of p21/Cip1. The relative promoter activity of p21/Cip1 enrichment by KLF4 normalized to IgG as a control was not changed (Figure 8B).

The HEK293a cells were co-transfected with plasmids containing human p21 promoter regions and human KLF4 plasmids. The overexpression of KLF4 did not upregulate p21 promoter activities (nucleotides: 5 kb to 30 bp) (Figure 8C) or p21 mRNA expression levels (Figure 8D). However, the results from the co-immunoprecipitation assay using anti-KLF4 and KLF4 transcription cofactor SMAD4 antibodies showed that knockdown of Myadm increased the KLF4-SMAD 4 complex formation, and hence upregulated the p21/Cip1 protein expression level. Overexpression of Myadm, on the other hand, reduced KLF4/SMAD4 complex formation and p21/Cip1 protein expression levels (Figure 8E).

Taken together, our results from the genome-wide search using ChIP-Seq, co- immunoprecipitation, and promoter activity detection suggest that Myadm affects KLF4/SMAD4 complex formation, which in turn regulates p21 protein expression through binding to its UTR.

## Discussion

In our present study, three major findings helped elucidate the significant role of miR-182-3p/Myadm/KLF4/p21 axis in PAH vascular remodeling. First, we showed that the reduction of miR-182-3p in plasma might be an indicator of PAH. Second, both loss-of-function and gain-of-function studies indicated that miR-182-3p could modulate PAH via targeting Myadm. Third, we observed that Myadm promoted the proliferation of PASMCs by inhibiting the KLF4 nuclear export-dependent p21 expression.

We first noticed that the deregulation of miR-182-3p expression level might be of prognostic significance in patients with PAH. This was consistent with previous studies showing the involvement of miRNAs in PAH. For example, the signature profile of miRNAs from mild-to-severe human PH subjects compared with the control subjects using miRNA arrays has recently reported elevated levels of mi-130a, mir-133b, miR-191, miR-204, and miR-208b in PH patients 25. In another study, plasma levels of miR-17, miR-21, and miR-190 in 40 healthy volunteers were found to be positively correlated with sPAP, which was examined during a high-altitude expedition up to an altitude of 7,050 m 26. In our present study, we showed that reduced plasma level of miR-182-3p was correlated with PAH both in patients and in animal models. This observation was confirmed by the inverse correlation between miR-182-3p expression level and pulmonary arterial systolic pressure.

In MCT- and hypoxia-induced PAH mice and rats, miR-182-3p could serve as a novel indicator for diagnosis and prognosis of the development and progression of PAH. Previously, decreased level of miR-182-3p was identified as a plasma biomarker for the diagnosis of malignant tumors 27. miR-182-3p has been suggested to be an indicator of high vascular remodeling risk because of its inhibition of SMC proliferation and migration and inverse correlation with the carotid neointimal formation both in animal models and patients 9. The current study has further extended our knowledge of the role of miR-182-3p and its target Myadm on vascular remodeling in PAH.

Our findings also suggest a promising potential of miR-182-3p for the treatment of PAH. This contention was supported by the observation that the reduction of miR-182-3p/overexpression of Myadm induced pulmonary vascular remodeling, and enhanced pulmonary artery blood flow resistance. Furthermore, miR-182-3p could reverse the MCT-induced pathological changes in hemodynamics and structure of the cardio-pulmonary system to nearly normal levels. In fact, miR-182-3p has previously been shown to have the therapeutic potential. For example, miR-182-3p exerted anti-inflammatory effects by negatively regulating on pro-inflammatory cytokine expression via targeting the TLR signaling pathway 28. In another instance, miR-182-3p was found to inhibit osteosarcoma by targeting EBF2 29. We have also identified the effect of miR-182-3p on carotid artery stenosis, another vascular remodeling disease 9. However, this is the first report of targeting vascular remodeling by miR-182-3p to treat PAH.

It is of note that the expression of the target of miR-182-3p, Myadm, a myeloid-differentiated marker, is not restricted to the hematopoietic system^30^. Recently, Myadm was shown to be involved in hypertension 31, confirming the correlation between Myadm and cardio-pulmonary diseases. In our present study, we found that Myadm was highly expressed throughout the lung including in the parenchyma and stroma, which is consistent with previous findings 32. Myadm was not unique to SMCs both in our present study and in previous studies, and was detected in macrophages, endothelial cells and epithelial cells of the lung 11, 33,34 .However, it remains unknown whether MCT-induced impairment in lung tissues activates myeloid-derived cells (such as macrophages) to differentiate into perivascular cells or directly mediates PASMC proliferation and migration by up-regulating Myadm. Our results indicate that Myadm-associated changes in PAH vascular remodeling are at least partially driven by increased PASMC proliferation and migration. However, in the future, it remains to be determined whether the effect of Myadm on vascular remodeling is also mediated by the inflammatory cells.

Another important finding from our study is that Myadm activates PASMC proliferation via the KLF4-dependent regulation of p21/Cip1 expression. KLF4 is a DNA-binding transcriptional regulator associated with growth arrest both in actively proliferating cells and in normal hematopoiesis 35. As a transcription factor, KLF4 is mainly present in the nucleus 36, 37. It has also been reported that under stress or influence of mitogens, KLF4 expression is induced, and it binds to the cis element on CDKN1A (the gene encoding p21Cip1/Waf1, a CDK1 inhibitor) increasing the promoter activity 38, 39. In our present study, Myadm knock-out blunted FBS-and PDGF-BB- induced PASMC proliferation via the up-regulated expression of p21/Cip1 through a KLF4 nuclear export-mediated mechanism without changing the total KLF4 expression level. This finding was supported by the evidence that Myadm promoted KLF4 translocation from the nucleus to the cytoplasm without changing the total expression level of KLF4 as evidenced by both Western blotting and immunofluorescence staining. Our results are consistent with the recent findings from various studies. For instance, Wamhoff et al. found that KLF4 localized to both cytoplasmic and nuclear compartments in SMCs undergoing phenotypic modulation 40. Also, Liu et al showed that PDGF-BB prompted the translocation of KLF4 to the cytoplasm through the CRM1-mediated nuclear export pathway in VSMCs and increased the interaction of KLF4 with actin in the cytoplasm triggering VSMC phenotype modulation, which favored proliferation and migration 41. One limitation of our study is that the precise mechanism of Myadm-induced nuclear export pathway of KLF4 is not known.

Although Myadm knockout increased the protein expression level of p21/Cip1 both in FBS- and PDGF-BB-treated PASMCs and in PAH rats, it did not change the promoter activity of p21/Cip1 as evidenced by the ChIP assay. Subsequent analysis with the next-generation sequencing technology (ChIP-seq) suggested that Myadm inhibited KLF4 binding to the intergenic regions of p21/Cip1. Hence, we speculated that Myadm accelerated the nuclear export of KLF4 followed by the reduction of the KLF4 binding to the UTR of p21/Cip1 leading to decreased p21/Cip1 protein expression. Our finding of the regulation of p21/Cip1 expression by KLF4 via the promoter activation independent pathway is substantiated by another study, which showed that PDGF-BB stimulated sumolyation of KLF4 that inhibited p21 transcription by recruiting transcriptional co-repressors to p21 promoter and thus enhanced VSMC proliferation 42. Furthermore, our results suggested that Myadm reduced KLF4/SMAD4 complex formation and p21/Cip1 protein expression levels. Whether Myadm-induced KLF4 nuclear export is associated with the reduction of KLF4/SMAD4 complex formation remains to be determined.

In summary, we have provided evidence that Myadm-induced PASMC proliferation plays a causal role in the development of PAH vascular remodeling via a KLF4 nuclear export-dependent mechanism, leading to a decreased p21/Cip1 expression level. In this context, miR-182-3p has the prognostic and therapeutic significance in PAH vascular remodeling. Most importantly, our results suggest that targeting miR-182-3p/Myadm/KLF4/p21 axis is a potential novel strategy for PAH treatment that should be explored in future preclinical and clinical studies.

## Figures and Tables

**Table 1:** oligonucleotide primers used for human KLF4 overexpression plasmid construction.

Primers	sequences
1	CTTGGTACCGAGCTCGGA
2	CTGACAGCCATGTCAGACTCGCCAGGTGGCTGCCTCATGGTGGCGGATCCGAGCTCGGTACCAAG
3	GAGTCTGACATGGCTGTCAGCGACGCGCTGCTCCCATCTTTCTCCACGTTCGCGTCTGGCCCGGC
4	CGCCAGCGGTTATTCGGGGCACCTGCTTGACGCAGTGTCTTCTCCCTTCCCGCCGGGCCAGACGC
5	CCGAATAACCGCTGGCGGGAGGAGCTCTCCCACATGAAGCGACTTCCCCCAGTGCTTCCCGGCCG
6	CGCCGCTCTCCAGGTCTGTGGCCACGGTCGCCGCCGCCAGGTCATAGGGGCGGCCGGGAAGCACT
7	GACCTGGAGAGCGGCGGAGCCGGTGCGGCTTGCGGCGGTAGCAACCTGGCGCCCCTACCTCGGAG
8	GGAGAGAATAAAGTCCAGGTCCAGGAGATCGTTGAACTCCTCGGTCTCTCTCCGAGGTAGGGGCG
9	GGACCTGGACTTTATTCTCTCCAATTCGCTGACCCATCCTCCGGAGTCAGTGGCCGCCACCGTGT
10	CAGGGCCGCTGCTCGACGGCGACGACGAAGAGGAGGCTGACGCTGACGAGGACACGGTGGCGGCC
11	CGAGCAGCGGCCCTGCCAGCGCGCCCTCCACCTGCAGCTTCACCTATCCGATCCGGGCCGGGAAC
12	TGCCATAGAGGAGGCCTCCGCCCGTGCCGCCCGGCGCCACGCCCGGGTCGTTCCCGGCCCGGATC
13	GAGGCCTCCTCTATGGCAGGGAGTCCGCTCCCCCTCCGACGGCTCCCTTCAACCTGGCGGACATC
14	GCCGCAGGAGCTCGGCCACGAAGCCGCCCGAGGGGCTCACGTCGTTGATGTCCGCCAGGTTGAAG
15	CCGAGCTCCTGCGGCCAGAATTGGACCCGGTGTACATTCCGCCGCAGCAGCCGCAGCCGCCAGGT
16	GCCAGGGGCGCTCAGCGACGCCTTCAGCACGAACTTGCCCATCAGCCCGCCACCTGGCGGCTGCG
17	CTGAGCGCCCCTGGCAGCGAGTACGGCAGCCCGTCGGTCATCAGCGTCAGCAAAGGCAGCCCTGA
18	CGCGGCGGCCCGCCGTTGTAGGGCGCCACCACCACCGGGTGGCTGCCGTCAGGGCTGCCTTTGCT
19	CGGGCCGCCGCGCACGTGCCCCAAGATCAAGCAGGAGGCGGTCTCTTCGTGCACCCACTTGGGCG
20	GGGAAGTCGTGTGCAGCCGGCCGGTGGCCATTGCTGAGAGGGGGTCCAGCGCCCAAGTGGGTGCA
21	GCTGCACACGACTTCCCCCTGGGGCGGCAGCTCCCCAGCAGGACTACCCCGACCCTGGGTCTTGA
22	GGAGGAAGCGGCAGGGCAGGGTGACAGTCCCTGCTGCTCAGCACTTCCTCAAGACCCAGGGTCGG
23	CCCTGCCGCTTCCTCCCGGCTTCCATCCCCACCCGGGGCCCAATTACCCATCCTTCCTGCCCGAT
24	CGGGACTGACCTTGGTAATGGAGCGGCGGGACTTGCGGCTGCATCTGATCGGGCAGGAAGGATGG
25	CATTACCAAGGTCAGTCCCGGGGATTTGTAGCTCGGGCTGGGGAGCCCTGTGTGTGCTGGCCCCA
26	CTCTAGGGGTGAAGAAGGTGGGGTGAGCATCATCCCGTGTGTCCCGAAGTGGGGCCAGCACACAC
27	CACCTTCTTCACCCCTAGAGCTCATGCCACCCGGTTCCTGCATGCCAGAGGAGCCCAAGCCAAAG
28	CAAGTGTGGGTGGCGGTCCTTTTCCGGGGCCACGATCGTCTTCCCCTCTTTGGCTTGGGCTCCTC
29	CCGCCACCCACACTTGTGATTACGCGGGCTGCGGCAAAACCTACACAAAGAGTTCCCATCTCAAG
30	CACAGTGGTAAGGTTTCTCACCTGTGTGGGTTCGCAGGTGTGCCTTGAGATGGGAACTCTTTGTG
31	GGTGAGAAACCTTACCACTGTGACTGGGACGGCTGTGGATGGAAATTCGCCCGCTCAGATGAACT
32	GGCACTGGAACGGGCGGTGCCCCGTGTGTTTACGGTAGTGCCTGGTCAGTTCATCTGAGCGGGCG
33	CGCCCGTTCCAGTGCCAAAAATGCGACCGAGCATTTTCCAGGTCGGACCACCTCGCCTTACACAT
34	TGCTGGATATCTGCAGAATTCTTAAAAATGCCTCTTCATGTGTAAGGCGAGGTGGT

**Table 2:** Primers for rat Myadm shRNA overexpression plasmid construction.

Target sequence	GTTTGGCGTATGCTACTGAAG
shRNA template S	5'AATTCGTTTGGCGTATGCTACTGAAGTTCAAGAGACTTCAGTAGCATACGCCAAACTTTTTTG3'
shRNA template A	5'GATCCAAAAAAGTTTGGCGTATGCTACTGAAGTCTCTTGAACTTCAGTAGCATACGCCAAACG3'
Transcription product	5'CGTTTGGCGTATGCTACTGAAGTTCAAGAGACTTCAGTAGCATACGCCAAACTTT3'

**Table 3:** Primers for recombinant adenovirus vector that overexpresses rat Myadm.

Target sequence	TTCTCCGAACGTGTCACGT
Control adenovirus S	5'AATTCGTTCTCCGAACGTGTCACGTTTCAAGAGAACGTGACACGTTCGGAGAACTTTTTTG3'
Control adenovirus A	GATCCAAAAAAGTTTGGCGTATGCTACTGAAGTCTCTTGAACTTCAGTAGCATACGCCAAACG
Transcription product	GTTCTCCGAACGTGTCACGTTTCAAGAGAACGTGACACGTTCGGAGAACTT

**Table 4:** Clinical Characteristics of PAH patient and control subjects

	Control	PAH
Age	58.09±10.73	55.46±14.19
Sex(Male/Female)	41/30	19/27
Height(cm)	163.35±9.13	165±8.06
Weight(Kg)	72.12±13.16	62.82±11.34
sPAP (mmHg)	NA	64.8±23.89
NYHA		
I	NA	1
II		12
III		23
IV		10
BNP (pg/mL)	NA	321.47
PAH drug therapy	0	2
miR-182-3p	2.34±0.92	0.74±0.56

sPAP: systolic pulmonary artery pressure; NYHA: cardiac function classification from New York Heart Association (NYHA); BNP: Brain natriuretic peptide; NA: not available.

## Abbreviations

Myadm: Myeloid-associated differentiation marker
PAH: pulmonary arterial hypertension
KLF4: Krüppel-like factor 4
PASMCs: pulmonary artery smooth muscle cells
PASP: pulmonary artery systolic pressure
RVSP: right ventricular systolic pressure
TAPSE: tricuspid annular displacement
PVmax: maximum pulmonary velocity
IMT: intimal-medial thickness
