# Supplementary material for: miR-182-3p/Myadm contribute to pulmonary artery hypertension vascular remodeling via a KLF4/p21-dependent mechanism
Source: Theranostics. 2020 Apr 25;10(12):5581–99. doi: 10.7150/thno.44687 (PMC7196306; doi:10.7150/thno.44687)
Supplement: Supplementary file 1 — Supplementary figures and tables. [file thnov10p5581s1.pdf]

Supplemental Figure and Figure Legends

Figure S1.

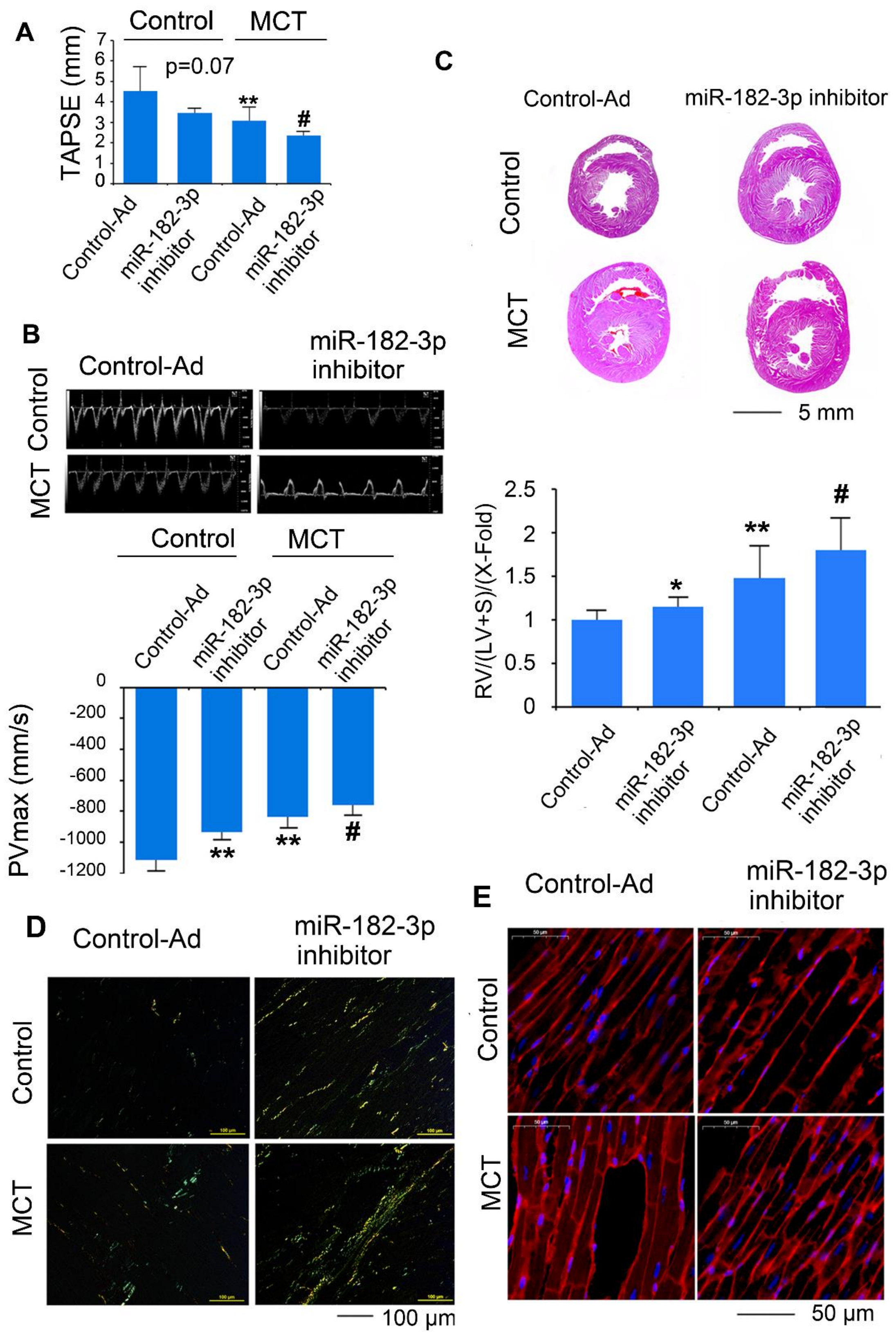

Figure S1. miR-182-3p inhibitor augments the effects of MCT on pulmonary hemodynamics change and cardiac remodeling in rats. Rats were transduced with either recombinant adenovirus expressing inhibitor (shRNA) against the miR-182-3p (miR-182-3p inhibitor ,10^{10} pfu/mL) or the control virus (Control-Ad) and then were injected subcutaneously using one dose of monocrotaline (MCT, 60 mg/kg body weight, normal saline was used as normal control). The tricuspid annular displacement (TAPSE; A) and pulmonary velocity maximum (PVmax; B) were detected using pulmonary pulsed-wave Doppler echocardiography. Whole hearts were embedded in paraffinum, sliced into 5-μm sections, and then hematoxylin and eosin (HE) staining was performed. The right ventricular hypertrophy (right ventricle/left ventricle + septum [RV/(LV+S)]) was measured (C). Sirius red (D) and AlexFluo-568 conjugated wheat germ agglutinin (E) staining was performed followed by examination with a light microscope (Nikon) were used to detect disorder of collagen deposition and myocardial fibrosis. The images are shown at 20×magnification and 40×magnification, respectively. Bar size were indicated in each picture. *$p<0.05$, **$p<0.01$ vs rats injected with control virus. #$p<0.05$, ##<0.01 vs rats treated with control virus and MCT. n=6-8 per group. n=6-8 each group.

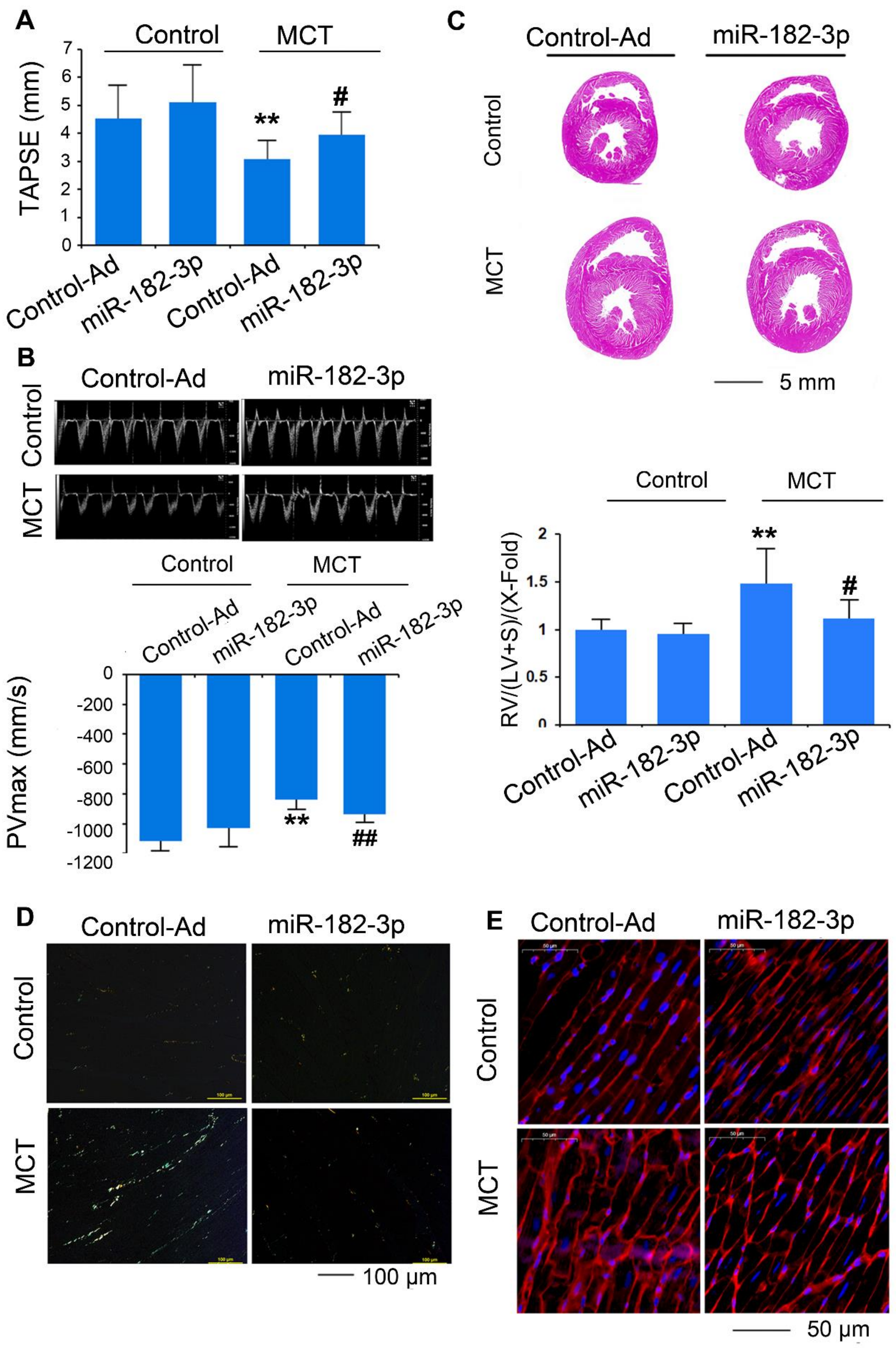

Figure S2. miR-182-3p regulates on pulmonary hemodynamics change and cardiac remodeling in MCT-induced PAH rats. Rats were transduced with either recombinant

adenovirus expresses the miR-182-3p (miR-182-3p,10^{10} pfu/mL) or the control virus (Control-Ad) and then were injected subcutaneously using one dose of MCT saline was used as normal control). The TAPSE (A) and PVmax (B) were detected using Doppler echocardiography. Whole hearts were embedded in paraffinum, sliced into 5-μm sections, and then the HE staining was performed. The right ventricular hypertrophy (right ventricle/left ventricle + septum [RV/(LV+S)]) were measured (C). Sirius red (D) and AlexFluo-568 conjugated wheat germ agglutinin (E) staining was performed followed by examination with a light microscope (Nikon) were used to detect disorder of collagen deposition and myocardial fibrosis. The images are shown at 20× and 40 ×magnification, respectively. Bar size were indicated in each picture. *$p<0.05$, **$p<0.01$ vs rats injected with control virus. #$p<0.05$, ##<0.01 vs rats treated with control virus and MCT. n=6-8 per group. n=6-8 each group.

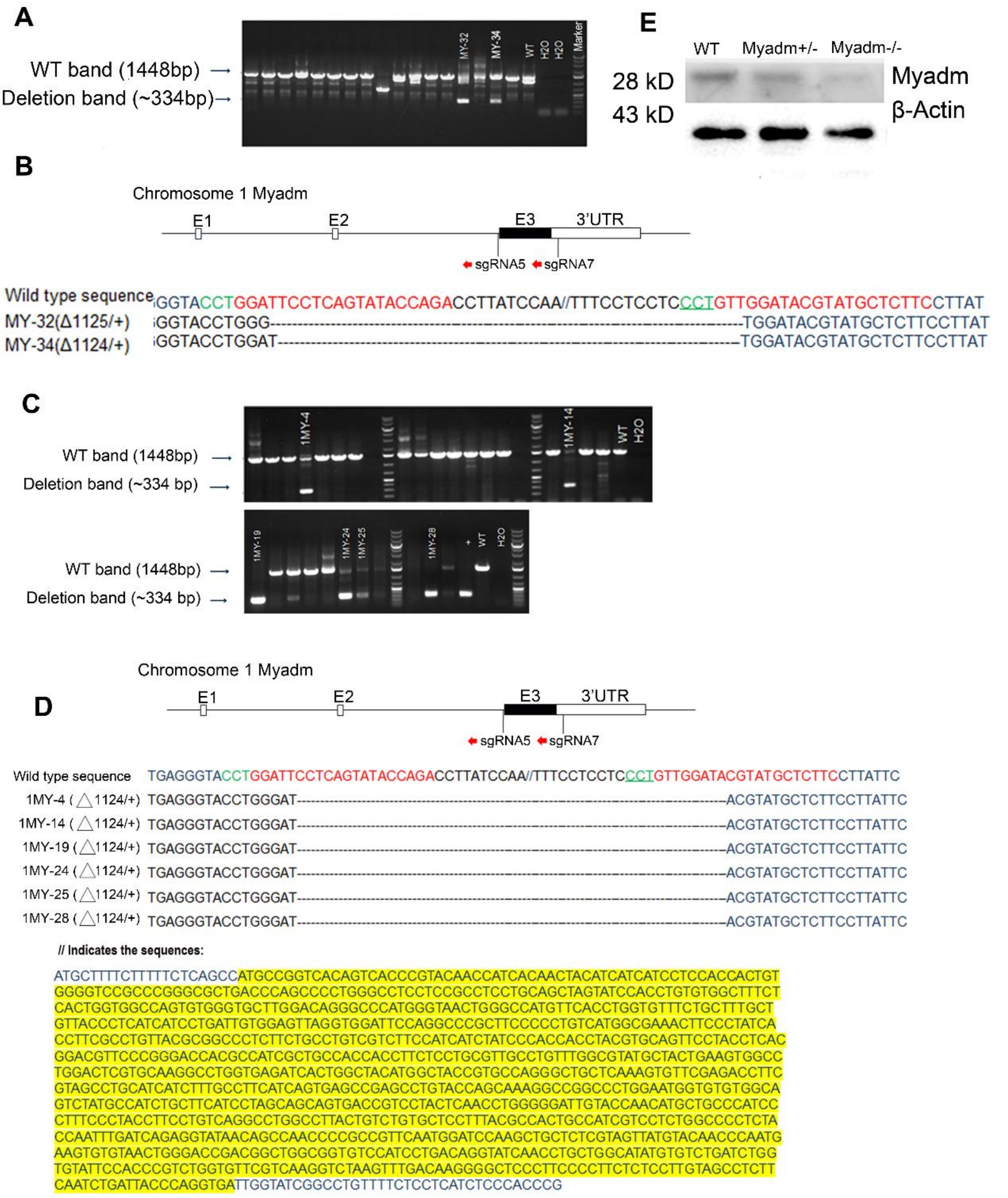

Figure S3. Identification of various Myadm genotype rats. Myadm$^{-/-}$ rats were generated and systematized using the CRISPR/Cas9 system. A. Agarose gel electrophoresis showing the PCR product of the target region derived from DNA isolated from the tails of MYADM knockout founder rats. Two out of 16 founder rats demonstrated site-specific sequence deletion (334 bp in length). B. Sequence analysis of the two founders at the target site. The sgRNA sequence is labelled in red, and the

PAM sequence is labelled in green. C. Agarose gel electrophoresis showing the PCR product of the target region derived from DNA isolated from the F1 hybrids. D. Sequence analysis of the $Myadm^{-/+}$ heterozygote at the target site. The sgRNA sequence is labeled in red, and the PAM sequence is labeled in green. E. The expression levels of Myadm in the lungs from the $Myadm^{+/+}$ (Wild-type), $Myadm^{+/-}$ (heterozygote) and Myadm knockout ($Myadm^{-/-}$) rats.

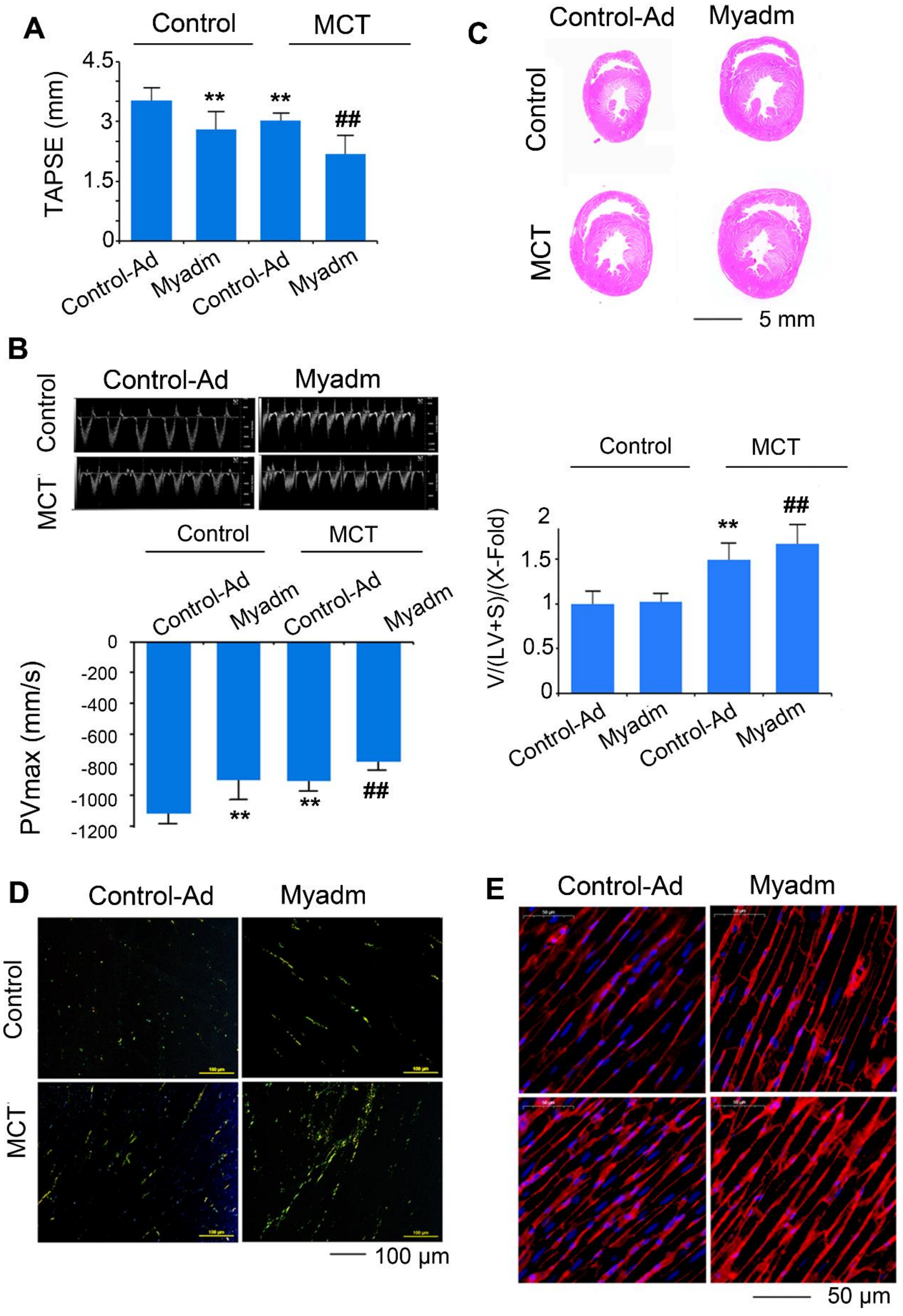

Figure S4. Myadm aggravates the pulmonary hemodynamics change and cardiac remodeling in MCT-induced PAH rats. Rats were transfected with either recombinant

adenovirus overexpresses Myadm gene (Myadm,10^{10} pfu/mL) or the control virus (Control-Ad) and then were injected subcutaneously using one dose of MCT (normal saline was used as normal control). The TAPSE (A) and PVmax (B) were detected using Doppler echocardiography. Whole hearts sections were embedded for HE staining, the right ventricular hypertrophy was measured (C). Sirius red (D) and AlexFluo-568 conjugated wheat germ agglutinin (E) staining were performed followed by examination with a light microscope (Nikon). The images are shown at 20× and 40× magnification, respectively. Bar size were indicated in each picture. *p<0.05, **p<0.01 vs rats injected with control virus. #p<0.05, ##<0.01 vs rats treated with control virus and MCT. n=6-8 per group. n=6-8 each group.

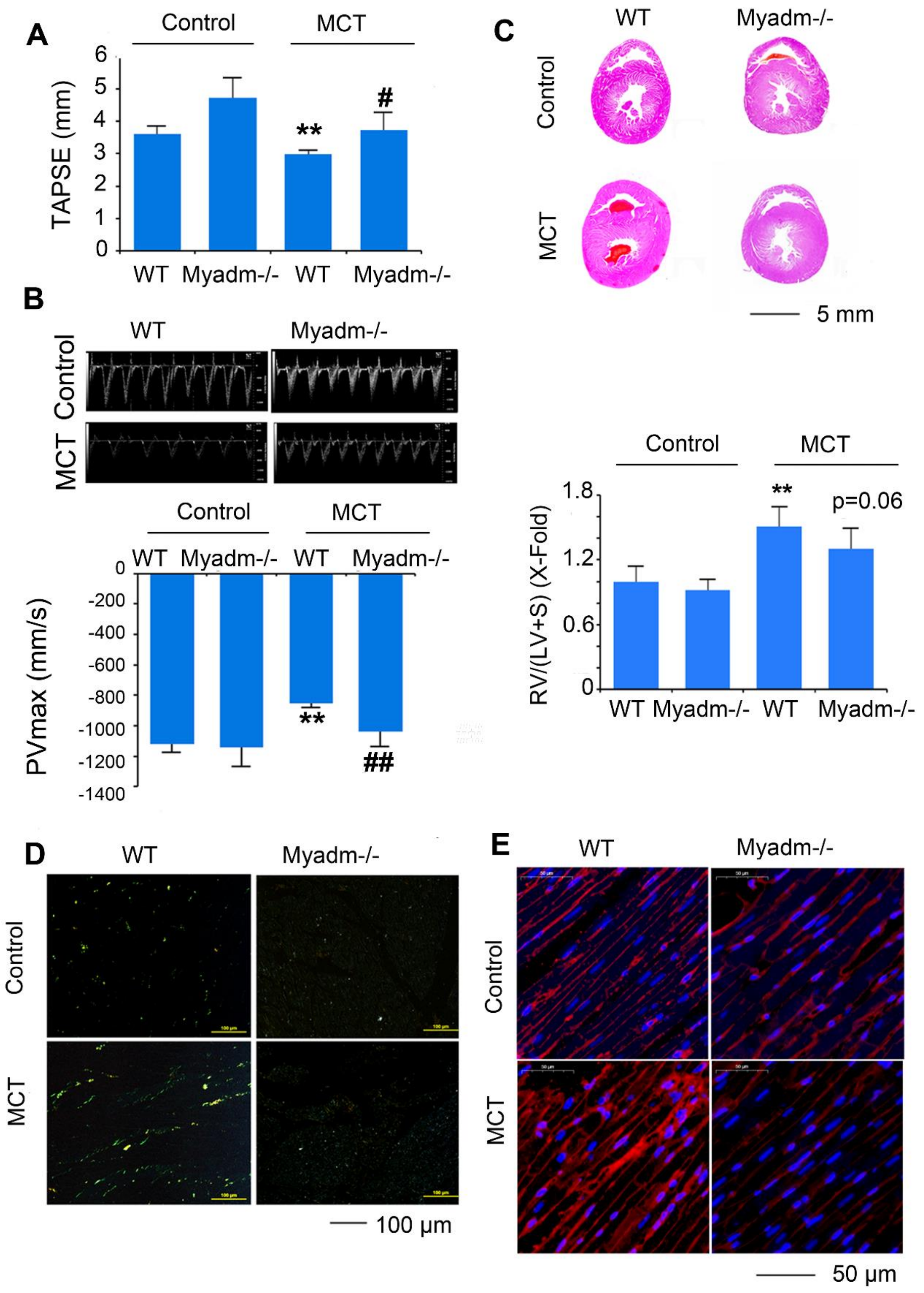

Figure S5. Knock down of Myadm inhibits pulmonary hemodynamics change and cardiac remodeling in MCT-induced PAH rats. Myadm$^{-/-}$ rats or the wild-type siblings of Myadm knockout rats (WT) were injected subcutaneously using MCT (normal

saline was used as normal control). The TAPSE (A) and the PVmax (B). Whole hearts were embedded in paraffinum, sliced into 5-μm sections followed by HE staining, the right ventricular hypertrophy was measured (C). Sirius red (D) and AlexFluo-568 conjugated wheat germ agglutinin (E) staining were performed followed by examination with a light microscope (Nikon) were used to detect disorder of collagen deposition and myocardial fibrosis. Bar size were indicated in each picture. n=6-8 each group. The images are shown at 20× and 40× magnification. Bar size were indicated in each picture. *$p<0.05$, **$p<0.01$ vs rats injected with or wild-type siblings of Myadm knockout rats. #$p<0.05$, ##<0.01 vs wild-type rats that injected with MCT. n=6-8 per group.

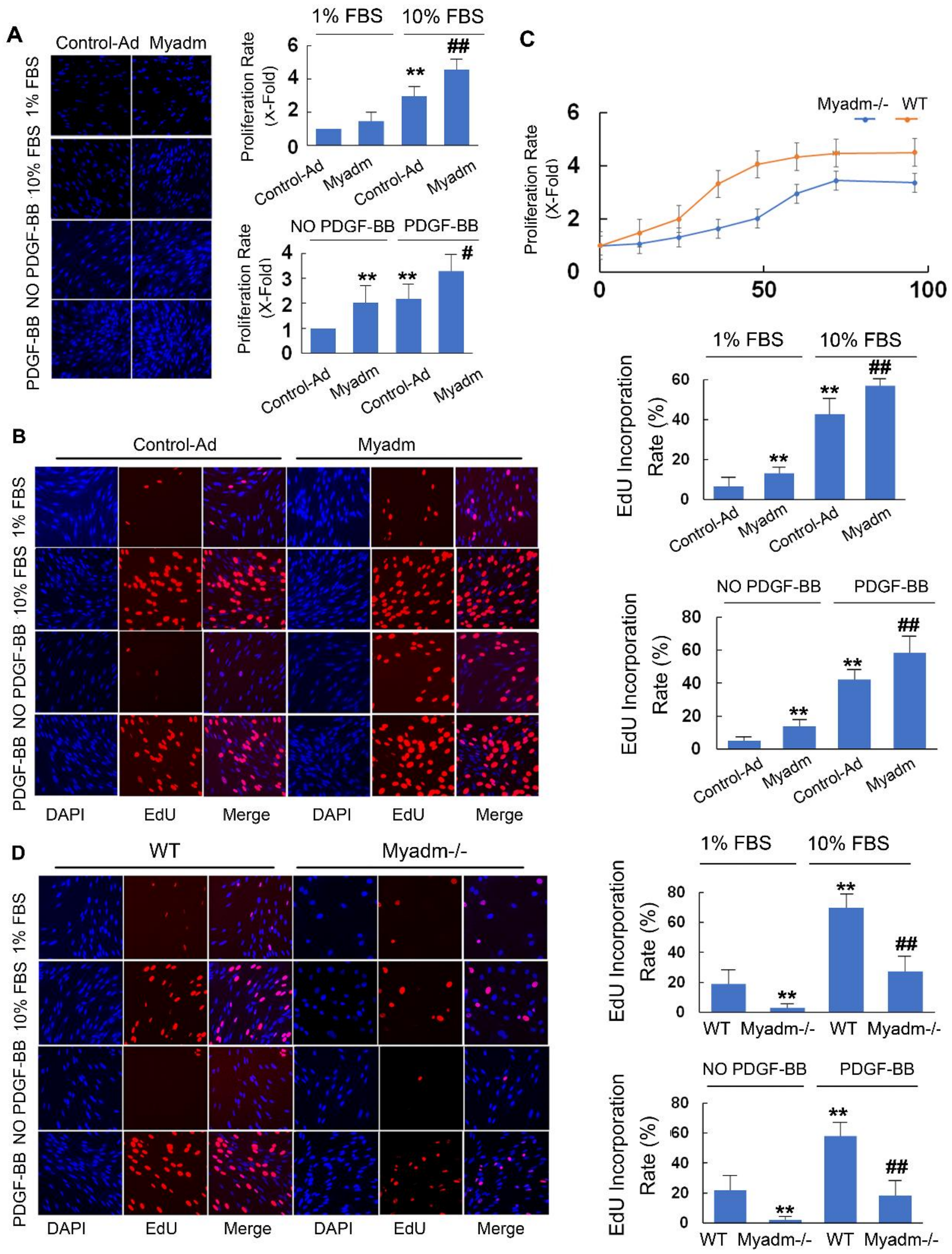

Figure S6. Myadm promotes PASMC proliferation. Quiescent rat PASMCs that overexpressed Myadm gene (Ad-Myadm) were then treated with stimulation with PDGF-BB (20 ng/mL) or the complete medium (10% FBS) for further 48 h. The cells were immediately fixed and permeabilized. A: the DAPI labeled cells were examined

using a Cellomics ArrayScan VTI HCS Reader. B: EdU incorporation assay was used to detect the DNA incorporation rate. Red fluorescent indicates the positive cells (DNA incorporation) and DAPI was employed to detect the nuclei. C: PASMCs isolated from the Myadm$^{-/-}$ rats were then treated with stimulation with PDGF-BB (20 ng/mL) or the complete medium (10% FBS) for further 48 h. CCK-8 staining was used according to the manufacturer guide to assess cell proliferation. D: EdU staining was determined according to the manufacturer's instructions. DAPI was employed to detect the nuclei. *p<0.05, **p<0.01 vs PASMCs infected with control virus or PASMCs isolated from wild-type siblings of Myadm knockout rats. #p<0.05, ##<0.01 vs PASMCs infected with control virus or PASMCs isolated from wild-type rats and in response to 10% FBS or PDGF-BB at a concentration of 20ng/mL. n= 6-8 in each group.

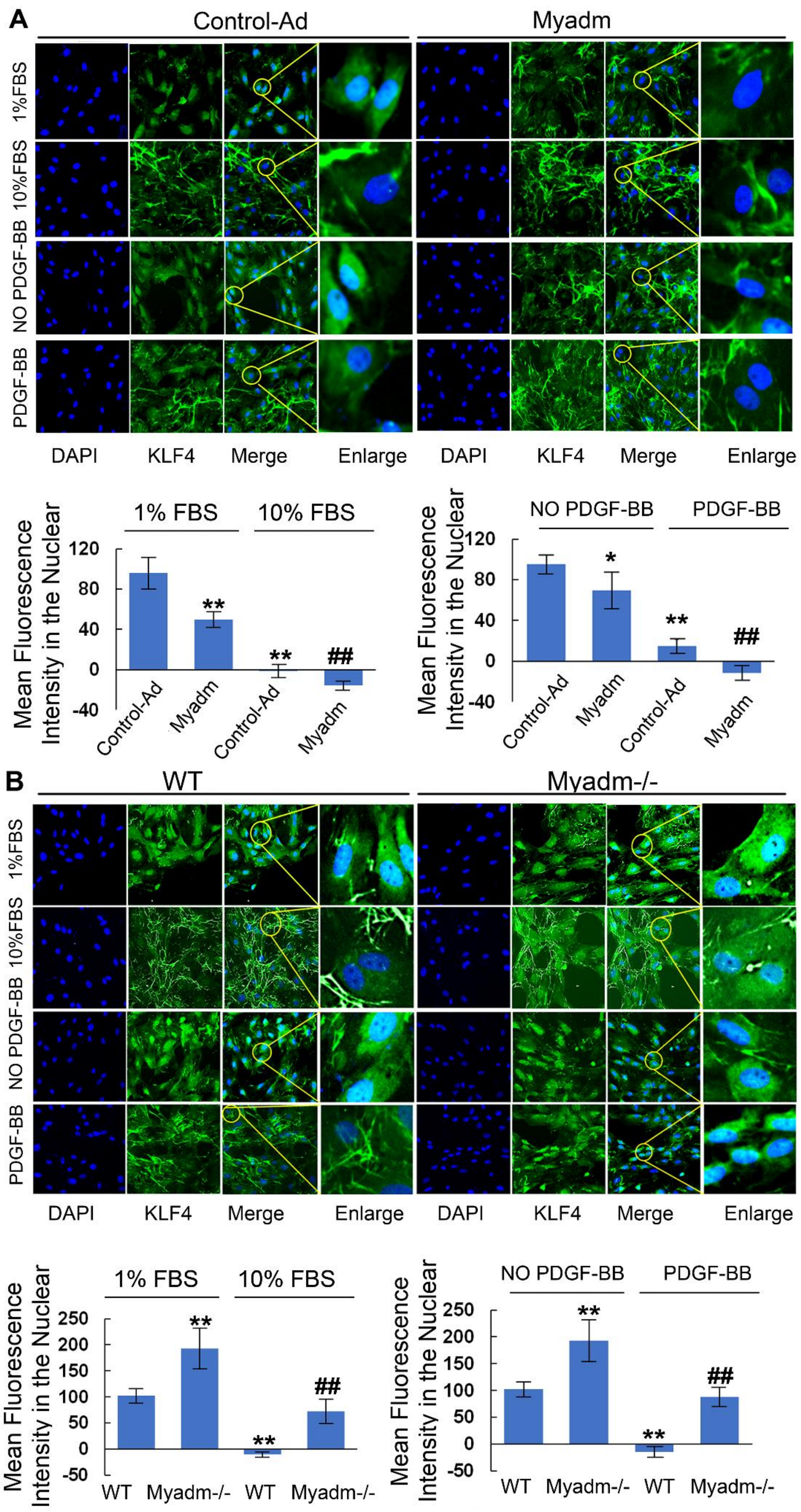

A
Control-Ad
Myadm
1%FBS
10%FBS
NO PDGF-BB
PDGF-BB
DAPI
KLF4
Merge
Enlarge
Mean Fluorescence Intensity in the Nuclear
1% FBS
10% FBS
NO PDGF-BB
Control-Ad
Myadm
**
##
*
B
WT
Myadm-/-

Figure S7. Myadm promotes PDGF-BB- and FBS-mediated KLF4 nuclear export. A: Quiescent rat PASMCs that overexpressed Myadm gene (Myadm) and B: PASMCs isolated from the $MYADM^{-/-}$ rats were then treated with stimulation with PDGF-BB (20 ng/mL) or complete medium (10% FBS) for further 48 h. KLF4 nuclear export was determined by the mean difference between nuclear and cytoplasmic immunocytofluorescence intensity. DAPI was employed to detect the nuclei. Data are expressed as X-fold induction compared to control. n=6-8 in each group. *p<0.05, **p<0.01 vs PASMCs infected with control virus or PASMCs isolated from wild-type siblings of Myadm knockout rats. #p<0.05, ##<0.01 vs PASMCs infected with control virus or PASMCs isolated from wild-type rats and in response to 10% FBS or PDGF-BB at a concentration of 20 ng/mL

Table S1. Differentially expressed proteins in the lungs of Myadm$^{-/-}$ rats compared to the control

Accession	Description	Ratio(Myadm$^{-/-}$/Myadm$^{+/-}$)	pValue
D3ZIA7	Anterior gradient 2 (Xenopus laevis) (Predicted), isoform CRA_a OS=Rattus norvegicus GN=Agr2 PE=4 SV=1 - [D3ZIA7_RAT]	0.084649	1.42E-27
Q9R1T1	Barrier-to-autointegration factor OS=Rattus norvegicus GN=Banf1 PE=1 SV=1 - [BAF_RAT]	10.317708	7.93E-21
Q05BA4	Myadm protein OS=Rattus norvegicus GN=Myadm PE=1 SV=1 - [Q05BA4_RAT]	0.144928	1.57E-17
Q6IFV1	Keratin, type I cytoskeletal 14 OS=Rattus norvegicus GN=Krt14 PE=2 SV=1 - [K1C14_RAT]	0.176887	2.01E-14
A0JPN3	BPI fold-containing family B member 1 OS=Rattus norvegicus GN=Bpifb1 PE=2 SV=1 - [BPIB1_RAT]	0.184116	7.71E-14
Q6IFV3	Keratin, type I cytoskeletal 15 OS=Rattus norvegicus GN=Krt15 PE=1 SV=1 - [K1C15_RAT]	0.207422	3.52E-12
F1LPK5	Acidic mammalian chitinase OS=Rattus norvegicus GN=Chia PE=3 SV=3 - [F1LPK5_RAT]	0.219924	2.08E-11
D3ZIA7	Anterior gradient 2 (Xenopus laevis) (Predicted), isoform CRA_a OS=Rattus norvegicus GN=Agr2 PE=4 SV=1 - [D3ZIA7_RAT]	4.962154	8.51E-11
Q6P6Q2	Keratin, type II cytoskeletal 5 OS=Rattus norvegicus GN=Krt5 PE=1 SV=1 - [K2C5_RAT]	0.233674	1.22E-10
D3ZJW6	RCG21066 OS=Rattus norvegicus GN=rCG_21066 PE=1 SV=1 - [D3ZJW6_RAT]	0.23736	1.91E-10
Q64315	Cyclin dependent kinase inhibitor OS=Rattus norvegicus GN=Cdkn1a PE=1 SV=1 - [Q64315_RAT]	4.804239	1.95E-10
A0A0G2K4I8	Uncharacterized protein OS=Rattus norvegicus PE=4 SV=1 - [A0A0G2K4I8_RAT]	0.249952	8.07E-10

D3ZAU0	Protein Muc5b OS=Rattus norvegicus GN=Muc5b PE=4 SV=2 - [D3ZAU0_RAT]	0.251052	9.09E-10
P11517	Hemoglobin subunit beta-2 OS=Rattus norvegicus PE=1 SV=2 - [HBB2_RAT]	0.254785	1.36E-09
A0A0G2JXF0	Uncharacterized protein OS=Rattus norvegicus PE=1 SV=1 - [A0A0G2JXF0_RAT]	0.25561	1.48E-09
Q6IFU8	Keratin, type I cytoskeletal 17 OS=Rattus norvegicus GN=Krt17 PE=1 SV=1 - [K1C17_RAT]	0.257617	1.83E-09
Q64232	Very-long-chain enoyl-CoA reductase OS=Rattus norvegicus GN=Tecr PE=1 SV=1 - [TECR_RAT]	0.263288	3.26E-09
M0RA79	Protein LOC691828 OS=Rattus norvegicus GN=LOC691828 PE=1 SV=1 - [M0RA79_RAT]	0.265426	4.03E-09
F1LZ11	Uncharacterized protein OS=Rattus norvegicus PE=1 SV=1 - [F1LZ11_RAT]	0.271519	7.27E-09
P01836	Ig kappa chain C region, A allele OS=Rattus norvegicus PE=1 SV=1 - [KACA_RAT]	0.272255	7.80E-09
D3ZJW6	RCG21066 OS=Rattus norvegicus GN=rCG_21066 PE=1 SV=1 - [D3ZJW6_RAT]	4.066216	1.09E-08
Q63011	Zero beta-globin (Fragment) OS=Rattus norvegicus PE=3 SV=1 - [Q63011_RAT]	0.278403	1.38E-08
Q68A21	Transcriptional activator protein Pur-beta OS=Rattus norvegicus GN=Purb PE=1 SV=3 - [PURB_RAT]	0.279851	1.57E-08
M0RD98	Uncharacterized protein OS=Rattus norvegicus PE=1 SV=2 - [M0RD98_RAT]	0.283356	2.15E-08
D3ZFF8	Uncharacterized protein OS=Rattus norvegicus PE=1 SV=2 - [D3ZFF8_RAT]	0.290648	4.02E-08
P01946	Hemoglobin subunit alpha-1/2 OS=Rattus norvegicus GN=Hba1 PE=1 SV=3 - [HBA_RAT]	0.292526	4.71E-08
B0K030	DnaJ (Hsp40) homolog, subfamily B, member 1 (Predicted), isoform CRA_b	3.805471	4.79E-08

	OS=Rattus norvegicus GN=Dnajb1 PE=1 SV=1 - [B0K030_RAT]		
O35543	Hematopoietic prostaglandin D synthase OS=Rattus norvegicus GN=Hpgds PE=1 SV=3 - [HPGDS_RAT]	0.29323	4.99E-08
P02091	Hemoglobin subunit beta-1 OS=Rattus norvegicus GN=Hbb PE=1 SV=3 - [HBB1_RAT]	0.293493	5.10E-08
P12346	Serotransferrin OS=Rattus norvegicus GN=Tf PE=1 SV=3 - [TRFE_RAT]	3.780362	5.54E-08
A0A0G2JWX0	Uncharacterized protein OS=Rattus norvegicus PE=4 SV=1 - [A0A0G2JWX0_RAT]	0.297106	6.86E-08
B0BNN3	Carbonic anhydrase 1 OS=Rattus norvegicus GN=Ca1 PE=1 SV=1 - [CAH1_RAT]	0.298765	7.84E-08
A0A0G2QC22	Protein RGD1306215 OS=Rattus norvegicus GN=RGD1306215 PE=1 SV=1 - [A0A0G2QC22_RAT]	3.687822	9.44E-08
P47967	Galectin-5 OS=Rattus norvegicus GN=Lgals5 PE=1 SV=2 - [LEG5_RAT]	0.302928	1.09E-07
Q63377	Sodium/potassium-transporting ATPase subunit beta-3 OS=Rattus norvegicus GN=Atp1b3 PE=2 SV=1 - [AT1B3_RAT]	3.630435	1.32E-07
A0JPN3	BPI fold-containing family B member 1 OS=Rattus norvegicus GN=Bpifb1 PE=2 SV=1 - [BPIB1_RAT]	3.602961	1.55E-07
Q5VLR6	BWK3 OS=Rattus norvegicus GN=LOC366772 PE=2 SV=1 - [Q5VLR6_RAT]	0.311273	2.06E-07
P07483	Fatty acid-binding protein, heart OS=Rattus norvegicus GN=Fabp3 PE=1 SV=2 - [FABPH_RAT]	3.365854	6.24E-07
P20761	Ig gamma-2B chain C region OS=Rattus norvegicus GN=Igh-1a PE=1 SV=1 - [IGG2B_RAT]	0.328794	7.14E-07
P20767	Ig lambda-2 chain C region OS=Rattus norvegicus PE=4 SV=1 - [LAC2_RAT]	0.329048	7.26E-07
A0A0G2JX64	Tropomyosin 1, alpha, isoform CRA_i OS=Rattus norvegicus GN=Tpm1 PE=1 SV=1 - [A0A0G2JX64_RAT]	3.306122	8.90E-07

P16290	Phosphoglycerate mutase 2 OS=Rattus norvegicus GN=Pgam2 PE=1 SV=2 - [PGAM2_RAT]	3.297342	9.37E-07
F2Z3T4	Muscleblind-like protein 2 OS=Rattus norvegicus GN=Mbnl2 PE=1 SV=1 - [MBNL2_RAT]	3.252585	1.22E-06
D3ZYE2	Protein RGD1565617 OS=Rattus norvegicus GN=RGD1565617 PE=1 SV=3 - [D3ZYE2_RAT]	0.337505	1.27E-06
D4AE31	Protein Fam126a OS=Rattus norvegicus GN=Fam126a PE=1 SV=1 - [D4AE31_RAT]	0.342995	1.79E-06
A0A0G2JSV6	Protein Hba-a2 OS=Rattus norvegicus GN=Hba-a2 PE=1 SV=1 - [A0A0G2JSV6_RAT]	0.351506	3.01E-06
Q4KM66	LOC500183 protein OS=Rattus norvegicus GN=LOC500183 PE=1 SV=1 - [Q4KM66_RAT]	0.351572	3.02E-06
D3ZAU0	Protein Muc5b OS=Rattus norvegicus GN=Muc5b PE=4 SV=2 - [D3ZAU0_RAT]	3.100742	3.05E-06
Q63041	Alpha-1-macroglobulin OS=Rattus norvegicus GN=A1m PE=1 SV=1 - [A1M_RAT]	0.351759	3.05E-06
F1M5L5	Protein Igkv11-125 OS=Rattus norvegicus GN=Igkv11-125 PE=1 SV=2 - [F1M5L5_RAT]	0.352096	3.11E-06
Q5M8A0	Kng2 protein OS=Rattus norvegicus GN=Kng2 PE=2 SV=1 - [Q5M8A0_RAT]	0.353369	3.36E-06
G3V7K1	Myomesin 2 OS=Rattus norvegicus GN=Myom2 PE=1 SV=1 - [G3V7K1_RAT]	3.059783	3.91E-06
A0A0G2K290	Uncharacterized protein OS=Rattus norvegicus PE=4 SV=1 - [A0A0G2K290_RAT]	0.361724	5.43E-06
P14141	Carbonic anhydrase 3 OS=Rattus norvegicus GN=Ca3 PE=1 SV=3 - [CAH3_RAT]	2.951841	7.53E-06
Q569B3	Igh-6 protein OS=Rattus norvegicus GN=Igh-6 PE=2 SV=1 - [Q569B3_RAT]	0.368578	7.93E-06
P04639	Apolipoprotein A-I OS=Rattus norvegicus GN=Apoa1 PE=1 SV=2 -	0.370637	8.87E-06

	[APOA1_RAT]		
F1LXW4	Protein Macc1 OS=Rattus norvegicus GN=Macc1 PE=1 SV=2 - [F1LXW4_RAT]	0.372594	9.84E-06
Q6MG90	Complement component 4, gene 2 OS=Rattus norvegicus GN=C4b PE=1 SV=1 - [Q6MG90_RAT]	0.374622	1.10E-05
D3ZCH9	Protein Cd177 OS=Rattus norvegicus GN=Cd177 PE=4 SV=2 - [D3ZCH9_RAT]	0.376777	1.23E-05
M0RBX3	Uncharacterized protein OS=Rattus norvegicus GN=Ighv1-9 PE=1 SV=2 - [M0RBX3_RAT]	0.377681	1.29E-05
Q9EPH1	Alpha-1B-glycoprotein OS=Rattus norvegicus GN=A1bg PE=2 SV=2 - [A1BG_RAT]	0.379597	1.42E-05
F1LYQ4	Uncharacterized protein OS=Rattus norvegicus GN=RGD1564515 PE=4 SV=2 - [F1LYQ4_RAT]	0.382328	1.63E-05
Q63011	Zero beta-globin (Fragment) OS=Rattus norvegicus PE=3 SV=1 - [Q63011_RAT]	2.821918	1.67E-05
Q6P6G4	2,3-bisphosphoglycerate mutase OS=Rattus norvegicus GN=Bpgm PE=1 SV=1 - [Q6P6G4_RAT]	0.389914	2.37E-05
A0A0G2JX36	Uncharacterized protein OS=Rattus norvegicus PE=1 SV=1 - [A0A0G2JX36_RAT]	0.390165	2.40E-05
A0A0A0MY22	Sialate O-acetylesterase OS=Rattus norvegicus GN=Siae PE=1 SV=1 - [A0A0A0MY22_RAT]	0.391154	2.52E-05
A0A0G2JXU4	Cytochrome P450 2A2 OS=Rattus norvegicus GN=Cyp2a3 PE=3 SV=1 - [A0A0G2JXU4_RAT]	2.733813	2.86E-05
Q9QX79	Fetuin-B OS=Rattus norvegicus GN=Fetub PE=2 SV=2 - [FETUB_RAT]	0.394102	2.90E-05
Q561R0	Marginal zone B- and B1-cell-specific protein OS=Rattus norvegicus GN=Mzb1 PE=2 SV=1 - [MZB1_RAT]	0.395342	3.08E-05
Q9EPH1	Alpha-1B-glycoprotein OS=Rattus norvegicus GN=A1bg PE=2 SV=2 - [A1BG_RAT]	2.72043	3.11E-05

Q5U329	Anion exchange protein OS=Rattus norvegicus GN=Slc4a1 PE=1 SV=1 - [Q5U329_RAT]	0.396301	3.22E-05
F1LTY5	Protein LOC100361009 OS=Rattus norvegicus GN=LOC100361052 PE=1 SV=3 - [F1LTY5_RAT]	0.396417	3.24E-05
A0A0H2UHQ8	40S ribosomal protein S17 OS=Rattus norvegicus GN=Rps17 PE=3 SV=1 - [A0A0H2UHQ8_RAT]	2.706831	3.38E-05
G3V885	Myosin-6 OS=Rattus norvegicus GN=Myh6 PE=1 SV=2 - [G3V885_RAT]	2.682111	3.94E-05
D3ZBB2	Uncharacterized protein OS=Rattus norvegicus PE=1 SV=3 - [D3ZBB2_RAT]	0.400626	3.94E-05
Q4G063	Cysteine-rich with EGF-like domain protein 2 OS=Rattus norvegicus GN=Creld2 PE=1 SV=1 - [CREL2_RAT]	0.401157	4.04E-05
G3V6G1	Immunoglobulin joining chain OS=Rattus norvegicus GN=Jchain PE=1 SV=1 - [G3V6G1_RAT]	0.40315	4.43E-05
F1LUS1	Uncharacterized protein OS=Rattus norvegicus PE=1 SV=2 - [F1LUS1_RAT]	0.403854	4.57E-05
Q4PP99	Cardiac troponin C OS=Rattus norvegicus GN=Tnnc1 PE=1 SV=1 - [Q4PP99_RAT]	2.602041	6.46E-05
Q5PQU1	Kininogen 1 OS=Rattus norvegicus GN=Kng1 PE=2 SV=1 - [Q5PQU1_RAT]	0.412084	6.59E-05
F1M663	Uncharacterized protein OS=Rattus norvegicus GN=Igkv5-37 PE=1 SV=2 - [F1M663_RAT]	0.412394	6.67E-05
Q6P734	Plasma protease C1 inhibitor OS=Rattus norvegicus GN=Serping1 PE=2 SV=1 - [IC1_RAT]	0.413139	6.89E-05
P08649	Complement C4 OS=Rattus norvegicus GN=C4 PE=1 SV=3 - [CO4_RAT]	0.415507	7.63E-05
Q63910	Alpha globin OS=Rattus norvegicus GN=Hba-a3 PE=1 SV=2 - [Q63910_RAT]	0.415584	7.66E-05
P08932	T-kininogen 2 OS=Rattus norvegicus PE=1 SV=2 - [KNT2_RAT]	0.417367	8.26E-05

Q62669	Protein Hbb-b1 OS=Rattus norvegicus GN=LOC103694855 PE=1 SV=1 - [Q62669_RAT]	0.42435	1.11E-04
F1LZH0	Protein LOC100912707 OS=Rattus norvegicus GN=LOC100912707 PE=1 SV=2 - [F1LZH0_RAT]	0.424603	1.12E-04
D3ZBS2	Inter-alpha-trypsin inhibitor heavy chain H3 OS=Rattus norvegicus GN=Itih3 PE=1 SV=3 - [D3ZBS2_RAT]	0.426647	1.21E-04
Q5VLR6	BWK3 OS=Rattus norvegicus GN=LOC366772 PE=2 SV=1 - [Q5VLR6_RAT]	2.461857	1.54E-04
P17209	Myosin light chain 4 OS=Rattus norvegicus GN=Myl4 PE=2 SV=2 - [MYL4_RAT]	2.458268	1.57E-04
F1M7K3	Myosin, light polypeptide 7, regulatory (Predicted), isoform CRA_a OS=Rattus norvegicus GN=Myl7 PE=4 SV=2 - [F1M7K3_RAT]	0.433707	1.61E-04
M0R789	Uncharacterized protein OS=Rattus norvegicus PE=4 SV=1 - [M0R789_RAT]	0.435193	1.70E-04
O35820	2'-deoxynucleoside 5'-phosphate N-hydrolase 1 OS=Rattus norvegicus GN=Dnph1 PE=1 SV=1 - [DNPH1_RAT]	0.437923	1.89E-04
P14046	Alpha-1-inhibitor 3 OS=Rattus norvegicus GN=A1i3 PE=1 SV=1 - [A1I3_RAT]	0.438875	1.96E-04
A0A0G2K0N6	Uncharacterized protein OS=Rattus norvegicus PE=1 SV=1 - [A0A0G2K0N6_RAT]	0.439093	1.98E-04
P02650	Apolipoprotein E OS=Rattus norvegicus GN=Apoe PE=1 SV=2 - [APOE_RAT]	0.441096	2.14E-04
Q5BJZ2	LOC367586 protein OS=Rattus norvegicus GN=LOC367586 PE=1 SV=1 - [Q5BJZ2_RAT]	0.44298	2.29E-04
A0A0G2JSJ1	Tumor-associated calcium signal transducer 2 OS=Rattus norvegicus GN=Tacstd2 PE=4 SV=1 - [A0A0G2JSJ1_RAT]	2.386525	2.46E-04
Q9WUW3	Complement factor I OS=Rattus	0.448625	2.83E-0

	norvegicus GN=Cfi PE=2 SV=1 - [CFAI_RAT]		4
Q03626	Murinoglobulin-1 OS=Rattus norvegicus GN=Mug1 PE=2 SV=1 - [MUG1_RAT]	0.45	2.97E-04
Q6MG74	B-factor, properdin OS=Rattus norvegicus GN=Cfb PE=2 SV=1 - [Q6MG74_RAT]	0.450485	3.02E-04
A0A0G2K684	Protein Cr2 OS=Rattus norvegicus GN=Cr2 PE=4 SV=1 - [A0A0G2K684_RAT]	2.347712	3.13E-04
Q01129	Decorin OS=Rattus norvegicus GN=Dcn PE=1 SV=1 - [PGS2_RAT]	2.346241	3.16E-04
A0A0G2JSH5	Serum albumin OS=Rattus norvegicus GN=Alb PE=1 SV=1 - [A0A0G2JSH5_RAT]	0.453737	3.40E-04
P02651	Apolipoprotein A-IV OS=Rattus norvegicus GN=Apoa4 PE=1 SV=2 - [APOA4_RAT]	0.456764	3.78E-04
G3V6D8	Myosin-3 OS=Rattus norvegicus GN=Myh3 PE=1 SV=1 - [G3V6D8_RAT]	2.316759	3.79E-04
F1LM19	Alpha-2-HS-glycoprotein OS=Rattus norvegicus GN=Ahsg PE=1 SV=3 - [F1LM19_RAT]	0.461235	4.42E-04
D3ZAZ0	Eukaryotic translation initiation factor 3 subunit M OS=Rattus norvegicus GN=Eif3m PE=1 SV=1 - [D3ZAZ0_RAT]	2.272541	4.98E-04
F1LWG8	5-hydroxytryptamine receptor 2B OS=Rattus norvegicus GN=Srl PE=1 SV=3 - [F1LWG8_RAT]	2.272206	4.99E-04
E9PT30	Protein Itln1 OS=Rattus norvegicus GN=Itln1 PE=4 SV=1 - [E9PT30_RAT]	2.265743	5.20E-04
D3ZPI8	Complement component 8, gamma polypeptide (Predicted), isoform CRA_a OS=Rattus norvegicus GN=C8g PE=1 SV=1 - [D3ZPI8_RAT]	0.466207	5.23E-04
A0A096P6L9	Complement C5 OS=Rattus norvegicus GN=C5 PE=1 SV=2 - [A0A096P6L9_RAT]	0.467391	5.44E-04
D3ZQ30	Protein Telo2 OS=Rattus norvegicus	0.471759	6.29E-0

	GN=Telo2 PE=1 SV=1 - [D3ZQ30_RAT]		4
A9YUA5	Cardiac troponin T2 OS=Rattus norvegicus GN=Tnnt2 PE=1 SV=1 - [A9YUA5_RAT]	2.232836	6.37E-04
P62332	ADP-ribosylation factor 6 OS=Rattus norvegicus GN=Arf6 PE=1 SV=2 - [ARF6_RAT]	2.218208	6.98E-04
B1WCA2	Tetratricopeptide repeat domain 25 OS=Rattus norvegicus GN=Ttc25 PE=2 SV=1 - [B1WCA2_RAT]	2.214286	7.15E-04
G3V6E7	Fibromodulin OS=Rattus norvegicus GN=Fmod PE=4 SV=1 - [G3V6E7_RAT]	0.478503	7.81E-04
Q5XIH1	Asporin OS=Rattus norvegicus GN=Aspn PE=1 SV=1 - [Q5XIH1_RAT]	2.199	7.86E-04
Q9ERM7	Secretory carrier membrane protein 2 (Fragment) OS=Rattus norvegicus GN=Scamp2 PE=2 SV=1 - [Q9ERM7_RAT]	0.479263	8.00E-04
Q9ET61	Complement component C1q receptor OS=Rattus norvegicus GN=Cd93 PE=1 SV=1 - [C1QR1_RAT]	2.191919	8.21E-04
F1LTN6	Protein Igkc OS=Rattus norvegicus GN=Igkc PE=1 SV=2 - [F1LTN6_RAT]	0.480358	8.28E-04
P07483	Fatty acid-binding protein, heart OS=Rattus norvegicus GN=Fabp3 PE=1 SV=2 - [FABPH_RAT]	0.48062	8.35E-04
B5DFG2	Hnrnpl protein (Fragment) OS=Rattus norvegicus GN=Hnrnpl PE=2 SV=1 - [B5DFG2_RAT]	2.184965	8.57E-04
Q04753	Methylosome subunit pICln OS=Rattus norvegicus GN=Clns1a PE=1 SV=1 - [ICLN_RAT]	0.481766	8.66E-04
D4A4K4	Protein Vps13c OS=Rattus norvegicus GN=Vps13c PE=1 SV=2 - [D4A4K4_RAT]	0.483019	9.00E-04
A0A0G2JZ75	Plasmalemma vesicle-associated protein OS=Rattus norvegicus GN=Plvap PE=4 SV=1 - [A0A0G2JZ75_RAT]	2.176636	9.03E-04
D3ZFC6	Protein Itih4 OS=Rattus norvegicus	0.483592	9.16E-0

	GN=Itih4 PE=1 SV=3 - [D3ZFC6_RAT]		4
M0RBP7	Uncharacterized protein OS=Rattus norvegicus PE=4 SV=1 - [M0RBP7_RAT]	0.485987	9.86E-04
D3ZFH5	Protein Itih2 OS=Rattus norvegicus GN=Itih2 PE=1 SV=3 - [D3ZFH5_RAT]	0.486034	9.88E-04
P12346	Serotransferrin OS=Rattus norvegicus GN=Tf PE=1 SV=3 - [TRFE_RAT]	0.486368	9.98E-04
D3ZEP5	Uncharacterized protein OS=Rattus norvegicus PE=1 SV=3 - [D3ZEP5_RAT]	0.486486	1.00E-03
F1LVL4	Uncharacterized protein OS=Rattus norvegicus PE=4 SV=2 - [F1LVL4_RAT]	0.487211	1.02E-03
B3DMA1	Atxn2l protein OS=Rattus norvegicus GN=Atxn2l PE=1 SV=1 - [B3DMA1_RAT]	0.489614	1.10E-03
A0A0G2JUY3	Uncharacterized protein OS=Rattus norvegicus PE=4 SV=1 - [A0A0G2JUY3_RAT]	0.491604	1.17E-03
A0A0A0MXW3	Histone H2A OS=Rattus norvegicus GN=H2afz PE=3 SV=1 - [A0A0A0MXW3_RAT]	2.107171	1.39E-03
D3Z9Z0	Ankyrin 1, erythroid OS=Rattus norvegicus GN=Ank1 PE=1 SV=1 - [D3Z9Z0_RAT]	0.499051	1.46E-03
G3V709	Nicotinate phosphoribosyltransferase OS=Rattus norvegicus GN=Naprt PE=1 SV=1 - [G3V709_RAT]	0.499512	1.47E-03
F1M9N5	Ubiquitin conjugation factor E4 A OS=Rattus norvegicus GN=Ube4a PE=1 SV=1 - [F1M9N5_RAT]	0.499698	1.48E-03
P14141	Carbonic anhydrase 3 OS=Rattus norvegicus GN=Ca3 PE=1 SV=3 - [CAH3_RAT]	0.503239	1.64E-03
P70623	Fatty acid-binding protein, adipocyte OS=Rattus norvegicus GN=Fabp4 PE=1 SV=3 - [FABP4_RAT]	0.503552	1.65E-03
A9YUA5	Cardiac troponin T2 OS=Rattus norvegicus GN=Tnnt2 PE=1 SV=1 - [A9YUA5_RAT]	0.503458	1.65E-03
Q62930	Complement component C9 OS=Rattus	0.503671	1.66E-0

	norvegicus GN=C9 PE=2 SV=1 - [CO9_RAT]		3
P23693	Troponin I, cardiac muscle OS=Rattus norvegicus GN=Tnni3 PE=1 SV=2 - [TNNI3_RAT]	2.075153	1.69E-03
Q8VID1	Dehydrogenase/reductase SDR family member 4 OS=Rattus norvegicus GN=Dhrs4 PE=2 SV=2 - [DHRS4_RAT]	0.5053	1.74E-03
F1LNH3	Procollagen, type VI, alpha 2, isoform CRA_a OS=Rattus norvegicus GN=Col6a2 PE=1 SV=2 - [F1LNH3_RAT]	2.069196	1.75E-03
M0RBJ7	Complement C3 OS=Rattus norvegicus GN=C3 PE=1 SV=1 - [M0RBJ7_RAT]	0.505855	1.77E-03
Q3KR94	Protein Vtn OS=Rattus norvegicus GN=Vtn PE=1 SV=1 - [Q3KR94_RAT]	0.506098	1.78E-03
P04638	Apolipoprotein A-II OS=Rattus norvegicus GN=Apoa2 PE=2 SV=1 - [APOA2_RAT]	0.506962	1.82E-03
D3ZVB7	Osteoglycin (Predicted) OS=Rattus norvegicus GN=Ogn PE=1 SV=1 - [D3ZVB7_RAT]	2.048193	1.99E-03
Q66H86	Olfactomedin-like protein 1 OS=Rattus norvegicus GN=Olfml1 PE=2 SV=1 - [OLFL1_RAT]	2.045889	2.02E-03
D3ZZC1	Protein Txndc5 OS=Rattus norvegicus GN=Txndc5 PE=1 SV=1 - [D3ZZC1_RAT]	0.510743	2.02E-03
P97569	Kallistatin OS=Rattus norvegicus PE=2 SV=1 - [P97569_RAT]	0.511945	2.09E-03
Q68FQ4	Uncharacterized protein C11orf70 homolog OS=Rattus norvegicus PE=2 SV=1 - [CK070_RAT]	0.514365	2.23E-03
A0A096MJZ2	Protein Tbl2 OS=Rattus norvegicus GN=Tbl2 PE=1 SV=1 - [A0A096MJZ2_RAT]	0.514894	2.26E-03
P20762	Ig gamma-2C chain C region OS=Rattus norvegicus PE=2 SV=1 - [IGG2C_RAT]	0.516297	2.35E-03
F1M3E9	Uncharacterized protein OS=Rattus norvegicus PE=4 SV=3 - [F1M3E9_RAT]	0.516297	2.35E-03

Q66HD3	Nuclear autoantigenic sperm protein OS=Rattus norvegicus GN=Nasp PE=1 SV=1 - [NASP_RAT]	2.018809	2.38E-03
A0A0G2JSP8	Creatine kinase M-type OS=Rattus norvegicus GN=Ckm PE=1 SV=1 - [A0A0G2JSP8_RAT]	2.01671	2.42E-03
Q68FY4	Group specific component OS=Rattus norvegicus GN=Gc PE=1 SV=1 - [Q68FY4_RAT]	0.519303	2.54E-03
E9PU10	Protein Ifi47 OS=Rattus norvegicus GN=Ifi47 PE=1 SV=3 - [E9PU10_RAT]	1.991525	2.82E-03
Q91ZN1	Coronin-1A OS=Rattus norvegicus GN=Coro1a PE=1 SV=3 - [COR1A_RAT]	1.991013	2.83E-03
P02764	Alpha-1-acid glycoprotein OS=Rattus norvegicus GN=Orm1 PE=2 SV=1 - [A1AG_RAT]	0.52381	2.86E-03
P31211	Corticosteroid-binding globulin OS=Rattus norvegicus GN=Serpina6 PE=1 SV=2 - [CBG_RAT]	0.525435	2.99E-03
A0A0G2K896	Protein RGD1310507 OS=Rattus norvegicus GN=RGD1310507 PE=1 SV=1 - [A0A0G2K896_RAT]	0.526439	3.06E-03
A0A0H2UHI5	Protein LOC299282 OS=Rattus norvegicus GN=Serpina3n PE=1 SV=1 - [A0A0H2UHI5_RAT]	0.526596	3.08E-03
F1M7K3	Myosin, light polypeptide 7, regulatory (Predicted), isoform CRA_a OS=Rattus norvegicus GN=Myl7 PE=4 SV=2 - [F1M7K3_RAT]	1.968047	3.25E-03
Q5U2U7	mRNA cap guanine-N7 methyltransferase OS=Rattus norvegicus GN=Rnmt PE=1 SV=1 - [MCES_RAT]	1.955631	3.51E-03
P17209	Myosin light chain 4 OS=Rattus norvegicus GN=Myl4 PE=2 SV=2 - [MYL4_RAT]	0.533447	3.65E-03
A0A0G2JSJ8	Fucosidase, alpha-L-1, tissue, isoform CRA_a OS=Rattus norvegicus GN=Fuca1 PE=1 SV=1 - [A0A0G2JSJ8_RAT]	0.536252	3.91E-03
Q6RUV5	Ras-related C3 botulinum toxin substrate 1 OS=Rattus norvegicus	1.935811	3.95E-03

	GN=Rac1 PE=1 SV=1 - [RAC1_RAT]		
D4A0A0	Dynein light chain roadblock-type 2 (Predicted) OS=Rattus norvegicus GN=Dynlrb2 PE=4 SV=1 - [D4A0A0_RAT]	0.539615	4.25E-03
D3ZBB8	Cytoplasmic dynein 2 heavy chain 1 OS=Rattus norvegicus GN=Dync2h1 PE=4 SV=3 - [D3ZBB8_RAT]	0.540074	4.29E-03
Q6AXR4	Beta-hexosaminidase subunit beta OS=Rattus norvegicus GN=Hexb PE=2 SV=1 - [HEXB_RAT]	0.540416	4.33E-03
Q6P775	Fibp protein OS=Rattus norvegicus GN=Fibp PE=1 SV=1 - [Q6P775_RAT]	1.917722	4.41E-03
D4A3Q2	Protein Stag1 OS=Rattus norvegicus GN=Stag1 PE=1 SV=1 - [D4A3Q2_RAT]	1.911894	4.57E-03
D3ZGM7	Protein Pi16 OS=Rattus norvegicus GN=Pi16 PE=1 SV=2 - [D3ZGM7_RAT]	1.911304	4.59E-03
P46844	Biliverdin reductase A OS=Rattus norvegicus GN=Blvra PE=1 SV=1 - [BIEA_RAT]	1.909497	4.64E-03
M0RBK4	Protein Igkv4-86 OS=Rattus norvegicus GN=Igkv4-86 PE=1 SV=2 - [M0RBK4_RAT]	0.543662	4.68E-03
B5DEK6	Cyhr1 protein OS=Rattus norvegicus GN=Cyhr1 PE=1 SV=1 - [B5DEK6_RAT]	0.544948	4.82E-03
M0R6T4	Protein Mms19 OS=Rattus norvegicus GN=Mms19 PE=1 SV=1 - [M0R6T4_RAT]	0.545198	4.85E-03
Q5XJW6	Cfh protein OS=Rattus norvegicus GN=Cfh PE=1 SV=1 - [Q5XJW6_RAT]	0.545455	4.88E-03
P14480	Fibrinogen beta chain OS=Rattus norvegicus GN=Fgb PE=1 SV=4 - [FIBB_RAT]	0.545455	4.88E-03
P04692	Tropomyosin alpha-1 chain OS=Rattus norvegicus GN=Tpm1 PE=1 SV=3 - [TPM1_RAT]	0.546737	5.03E-03
P83868	Prostaglandin E synthase 3 OS=Rattus norvegicus GN=Ptges3 PE=1 SV=2 - [TEBP_RAT]	1.895349	5.05E-03

Q794E4	Heterogeneous nuclear ribonucleoprotein F OS=Rattus norvegicus GN=Hnrnpf PE=1 SV=3 - [HNRPF_RAT]	1.893037	5.12E-03
Q5RJQ4	NAD-dependent protein deacetylase sirtuin-2 OS=Rattus norvegicus GN=Sirt2 PE=1 SV=1 - [SIR2_RAT]	0.547561	5.13E-03
P62275	40S ribosomal protein S29 OS=Rattus norvegicus GN=Rps29 PE=1 SV=2 - [RS29_RAT]	1.887244	5.31E-03
D4A9W3	Protein RGD1311756 OS=Rattus norvegicus GN=RGD1311756 PE=1 SV=1 - [D4A9W3_RAT]	0.549763	5.40E-03
B1PLB2	CD34 antigen isoform 2 OS=Rattus norvegicus GN=Cd34 PE=2 SV=1 - [B1PLB2_RAT]	1.883844	5.42E-03
Q01177	Plasminogen OS=Rattus norvegicus GN=Plg PE=2 SV=2 - [PLMN_RAT]	0.550349	5.47E-03
F1LYQ8	FERM, RhoGEF and pleckstrin domain-containing protein 1 OS=Rattus norvegicus GN=Farp1 PE=1 SV=2 - [FARP1_RAT]	0.551351	5.60E-03
D3ZN95	Protein Hcfc1 OS=Rattus norvegicus GN=Hcfc1 PE=1 SV=1 - [D3ZN95_RAT]	1.874494	5.73E-03
Q5U2T5	Protein Stard3 OS=Rattus norvegicus GN=Stard3 PE=1 SV=1 - [Q5U2T5_RAT]	0.55249	5.74E-03
Q5XIM5	Protein CDV3 homolog OS=Rattus norvegicus GN=Cdv3 PE=1 SV=1 - [CDV3_RAT]	1.873896	5.75E-03
Q6AYT5	Protein-glutamate O-methyltransferase OS=Rattus norvegicus GN=Armt1 PE=1 SV=1 - [ARMT1_RAT]	1.872754	5.79E-03
P07895	Superoxide dismutase [Mn], mitochondrial OS=Rattus norvegicus GN=Sod2 PE=1 SV=2 - [SODM_RAT]	1.871002	5.85E-03
Q9EST6	Acidic leucine-rich nuclear phosphoprotein 32 family member B OS=Rattus norvegicus GN=Anp32b PE=1 SV=1 - [AN32B_RAT]	1.868395	5.94E-03
G3V8D4	Apolipoprotein C-II (Predicted) OS=Rattus norvegicus GN=Apoc2	0.554096	5.96E-03

	PE=1 SV=1 - [G3V8D4_RAT]		
Q5XIA5	Coenzyme A synthase OS=Rattus norvegicus GN=Coasy PE=1 SV=1 - [Q5XIA5_RAT]	0.555247	6.11E-03
P42123	L-lactate dehydrogenase B chain OS=Rattus norvegicus GN=Ldhb PE=1 SV=2 - [LDHB_RAT]	1.86236	6.16E-03
D4A5L9	Protein LOC679794 OS=Rattus norvegicus GN=LOC679794 PE=3 SV=1 - [D4A5L9_RAT]	1.854305	6.47E-03
A0A0G2K9Y5	Histidine-rich glycoprotein OS=Rattus norvegicus GN=Hrg PE=1 SV=1 - [A0A0G2K9Y5_RAT]	0.557845	6.48E-03
A0A096MKD4	Protein Ldb3 OS=Rattus norvegicus GN=Ldb3 PE=1 SV=1 - [A0A096MKD4_RAT]	0.55819	6.53E-03
Q63468	Phosphoribosyl pyrophosphate synthase-associated protein 1 OS=Rattus norvegicus GN=Prpsap1 PE=1 SV=1 - [KPRA_RAT]	0.558424	6.56E-03
Q6AYT7	Monoacylglycerol lipase ABHD12 OS=Rattus norvegicus GN=Abhd12 PE=2 SV=1 - [ABD12_RAT]	0.559211	6.68E-03
B2RYM3	Inter-alpha trypsin inhibitor, heavy chain 1 (Predicted), isoform CRA_a OS=Rattus norvegicus GN=Itih1 PE=1 SV=1 - [B2RYM3_RAT]	0.55942	6.71E-03
D3ZR64	Protein Zfp598 OS=Rattus norvegicus GN=Zfp598 PE=1 SV=2 - [D3ZR64_RAT]	0.560088	6.81E-03
P41562	Isocitrate dehydrogenase [NADP] cytoplasmic OS=Rattus norvegicus GN=Idh1 PE=1 SV=1 - [IDHC_RAT]	1.841751	6.98E-03
A0A0G2JY31	Alpha-1-antiproteinase OS=Rattus norvegicus GN=Serpina1 PE=1 SV=1 - [A0A0G2JY31_RAT]	0.56124	6.98E-03
O35520	5I2 antigen (Fragment) OS=Rattus norvegicus GN=Cr1l PE=2 SV=1 - [O35520_RAT]	1.835443	7.24E-03
Q8CFN2	Cell division control protein 42 homolog OS=Rattus norvegicus GN=Cdc42 PE=1 SV=2 - [CDC42_RAT]	1.834208	7.30E-03
A0A0A0MY0	Endoplasmin OS=Rattus norvegicus	0.564589	7.51E-0

9	GN=Hsp90b1 PE=1 SV=1 - [A0A0A0MY09_RAT]		3
P02767	Transthyretin OS=Rattus norvegicus GN=Ttr PE=1 SV=1 - [TTHY_RAT]	0.564617	7.52E-03
Q641X3	Beta-hexosaminidase subunit alpha OS=Rattus norvegicus GN=Hexa PE=2 SV=1 - [HEXA_RAT]	0.565345	7.64E-03
Q08290	Calponin-1 OS=Rattus norvegicus GN=Cnn1 PE=1 SV=1 - [CNN1_RAT]	1.825994	7.67E-03
Q04631	Protein farnesyltransferase/geranylgeranyltransferase type-1 subunit alpha OS=Rattus norvegicus GN=Fnta PE=1 SV=1 - [FNTA_RAT]	0.566194	7.78E-03
Q63118	Calcitonin gene-related peptide type 1 receptor OS=Rattus norvegicus GN=Calcrl PE=2 SV=1 - [CALRL_RAT]	1.819136	7.99E-03
Q4V7D1	Signal sequence receptor, alpha OS=Rattus norvegicus GN=Ssr1 PE=1 SV=1 - [Q4V7D1_RAT]	0.569787	8.39E-03
Q3MHS7	GDP-mannose 4, 6-dehydratase OS=Rattus norvegicus GN=Gmds PE=2 SV=1 - [Q3MHS7_RAT]	0.571804	8.76E-03
B5DF79	Rnaset2 protein OS=Rattus norvegicus GN=Rnaset2 PE=2 SV=1 - [B5DF79_RAT]	0.572442	8.88E-03
B0BNC0	Ckmt2 protein OS=Rattus norvegicus GN=Ckmt2 PE=2 SV=1 - [B0BNC0_RAT]	0.57304	8.99E-03
D4ACV3	Histone H2A OS=Rattus norvegicus GN=Hist2h2ac PE=3 SV=2 - [D4ACV3_RAT]	1.792103	9.38E-03
P30152	Neutrophil gelatinase-associated lipocalin OS=Rattus norvegicus GN=Lcn2 PE=1 SV=2 - [NGAL_RAT]	1.786145	9.72E-03
P47820	Angiotensin-converting enzyme OS=Rattus norvegicus GN=Ace PE=1 SV=1 - [ACE_RAT]	1.784716	9.80E-03
P29419	ATP synthase subunit e, mitochondrial OS=Rattus norvegicus GN=Atp5i PE=1 SV=3 - [ATP5I_RAT]	1.777886	1.02E-02
O08651	D-3-phosphoglycerate dehydrogenase OS=Rattus norvegicus GN=Phgdh	0.578984	1.02E-02

	PE=1 SV=3 - [SERA_RAT]		
F1LQQ8	Beta-glucuronidase OS=Rattus norvegicus GN=Gusb PE=1 SV=2 - [F1LQQ8_RAT]	0.580196	1.04E-02
G3V6E7	Fibromodulin OS=Rattus norvegicus GN=Fmod PE=4 SV=1 - [G3V6E7_RAT]	1.772727	1.05E-02
P02680	Fibrinogen gamma chain OS=Rattus norvegicus GN=Fgg PE=1 SV=3 - [FIBG_RAT]	0.580542	1.05E-02
Q2XTA8	NADH dehydrogenase 1 beta 4 (Fragment) OS=Rattus norvegicus GN=Ndufb4 PE=2 SV=1 - [Q2XTA8_RAT]	0.580558	1.05E-02
Q5D005	alpha-1,2-Mannosidase (Fragment) OS=Rattus norvegicus GN=Man1a1 PE=2 SV=1 - [Q5D005_RAT]	0.581315	1.06E-02
Q5U3Z7	Serine hydroxymethyltransferase OS=Rattus norvegicus GN=Shmt2 PE=1 SV=1 - [Q5U3Z7_RAT]	0.581505	1.07E-02
F1M589	Protein Pml OS=Rattus norvegicus GN=Pml PE=1 SV=3 - [F1M589_RAT]	1.767651	1.08E-02
B1H282	Glycosyltransferase 25 domain containing 1 OS=Rattus norvegicus GN=Colgalt1 PE=1 SV=1 - [B1H282_RAT]	0.582386	1.09E-02
D4A2F6	Protein Nhlrc3 OS=Rattus norvegicus GN=Nhlrc3 PE=1 SV=2 - [D4A2F6_RAT]	0.582996	1.10E-02
P23928	Alpha-crystallin B chain OS=Rattus norvegicus GN=Cryab PE=1 SV=1 - [CRYAB_RAT]	1.763411	1.11E-02
B5DFK8	Pdxdc1 protein OS=Rattus norvegicus GN=Pdxdc1 PE=2 SV=1 - [B5DFK8_RAT]	0.583573	1.11E-02
P20059	Hemopexin OS=Rattus norvegicus GN=Hpx PE=1 SV=3 - [HEMO_RAT]	0.584256	1.13E-02
Q6AYD5	G1 to S phase transition 1 OS=Rattus norvegicus GN=Gspt1 PE=1 SV=1 - [Q6AYD5_RAT]	0.586047	1.17E-02
A0A0G2K2D9	Uncharacterized protein OS=Rattus norvegicus PE=1 SV=1 - [A0A0G2K2D9_RAT]	0.58596	1.17E-02

Q3KRE2	Methyltransferase like 7A OS=Rattus norvegicus GN=Mettl7a PE=1 SV=1 - [Q3KRE2_RAT]	1.74583	1.23E-02
P06757	Alcohol dehydrogenase 1 OS=Rattus norvegicus GN=Adh1 PE=1 SV=3 - [ADH1_RAT]	0.58866	1.23E-02
D3ZZT9	Protein Col14a1 OS=Rattus norvegicus GN=Col14a1 PE=1 SV=3 - [D3ZZT9_RAT]	0.591195	1.29E-02
P50393	Cytosolic phospholipase A2 OS=Rattus norvegicus GN=Pla2g4a PE=1 SV=1 - [PA24A_RAT]	0.591813	1.31E-02
P01015	Angiotensinogen OS=Rattus norvegicus GN=Agt PE=1 SV=1 - [ANGT_RAT]	0.592145	1.32E-02
D4A678	Protein Spta1 OS=Rattus norvegicus GN=Spta1 PE=1 SV=3 - [D4A678_RAT]	0.592807	1.33E-02
A0A0G2K7X0	Protein LOC100909983 OS=Rattus norvegicus GN=LOC100909983 PE=4 SV=1 - [A0A0G2K7X0_RAT]	1.729934	1.35E-02
G3V8L1	PYD and CARD domain containing OS=Rattus norvegicus GN=Pycard PE=1 SV=1 - [G3V8L1_RAT]	0.593935	1.36E-02
A0A0G2JT00	Divalent cation tolerant protein CUTA, isoform CRA_c OS=Rattus norvegicus GN=Cuta PE=1 SV=1 - [A0A0G2JT00_RAT]	0.594286	1.37E-02
A0A0G2K6H2	Maleylacetoacetate isomerase OS=Rattus norvegicus GN=Gstz1 PE=1 SV=1 - [A0A0G2K6H2_RAT]	0.595092	1.39E-02
A0A096MJW5	Phosphoinositide phospholipase C OS=Rattus norvegicus GN=Plch1 PE=4 SV=1 - [A0A096MJW5_RAT]	0.595209	1.40E-02
D3ZWD6	Complement component 8, alpha polypeptide (Predicted) OS=Rattus norvegicus GN=C8a PE=1 SV=1 - [D3ZWD6_RAT]	0.595388	1.40E-02
Q5PPN5	Tubulin polymerization-promoting protein family member 3 OS=Rattus norvegicus GN=Tppp3 PE=2 SV=1 - [TPPP3_RAT]	1.717268	1.46E-02
B0BNI5	Olfactomedin-like protein 3 OS=Rattus norvegicus GN=Olfml3 PE=2 SV=2 -	1.716004	1.47E-02

	[OLFL3_RAT]		
A0A0G2JYK0	Protein LOC299282 OS=Rattus norvegicus GN=LOC299282 PE=1 SV=1 - [A0A0G2JYK0_RAT]	0.599165	1.50E-02
G3V9Q3	Heterogeneous nuclear ribonucleoprotein H OS=Rattus norvegicus GN=Hnrnph1 PE=1 SV=2 - [G3V9Q3_RAT]	1.711066	1.51E-02
Q9QZ76	Myoglobin OS=Rattus norvegicus GN=Mb PE=1 SV=3 - [MYG_RAT]	0.599879	1.52E-02
P62329	Thymosin beta-4 OS=Rattus norvegicus GN=Tmsb4x PE=1 SV=2 - [TYB4_RAT]	1.708817	1.53E-02
B0BND9	Protein Srp72 OS=Rattus norvegicus GN=Srp72 PE=1 SV=1 - [B0BND9_RAT]	1.707707	1.54E-02
G3V878	Signal peptidase complex catalytic subunit SEC11 OS=Rattus norvegicus GN=Sec11c PE=1 SV=1 - [G3V878_RAT]	0.601643	1.57E-02
P23693	Troponin I, cardiac muscle OS=Rattus norvegicus GN=Tnni3 PE=1 SV=2 - [TNNI3_RAT]	0.601634	1.57E-02
P63045	Vesicle-associated membrane protein 2 OS=Rattus norvegicus GN=Vamp2 PE=1 SV=2 - [VAMP2_RAT]	0.601966	1.58E-02
Q9R1T3	Cathepsin Z OS=Rattus norvegicus GN=Ctsz PE=1 SV=2 - [CATZ_RAT]	0.602557	1.60E-02
Q5BJP7	Afm protein (Fragment) OS=Rattus norvegicus GN=Afm PE=2 SV=1 - [Q5BJP7_RAT]	0.602564	1.60E-02
D4A7J8	PRP4 pre-mRNA processing factor 4 homolog (Yeast) OS=Rattus norvegicus GN=Prpf4 PE=1 SV=1 - [D4A7J8_RAT]	1.700104	1.61E-02
Q01579	Glutathione S-transferase theta-1 OS=Rattus norvegicus GN=Gstt1 PE=1 SV=2 - [GSTT1_RAT]	1.69891	1.62E-02
A7LNF8	MHC class I antigen (Fragment) OS=Rattus norvegicus GN=RT1.A PE=2 SV=1 - [A7LNF8_RAT]	1.69781	1.63E-02
D4A5S9	Protein Prpf39 OS=Rattus norvegicus GN=Prpf39 PE=1 SV=1 - [D4A5S9_RAT]	0.603604	1.63E-02

Q5EBD4	Dcps protein (Fragment) OS=Rattus norvegicus GN=Dcps PE=2 SV=1 - [Q5EBD4_RAT]	1.697674	1.64E-02
Q5U1Y2	Protein Rac2 OS=Rattus norvegicus GN=Rac2 PE=1 SV=1 - [Q5U1Y2_RAT]	1.695351	1.66E-02
B5DEH4	Protein Uap1l1 OS=Rattus norvegicus GN=Uap1l1 PE=1 SV=1 - [B5DEH4_RAT]	0.604966	1.67E-02
B5DF57	Erythrocyte protein band 4.2 OS=Rattus norvegicus GN=Epb42 PE=1 SV=1 - [B5DF57_RAT]	0.605128	1.68E-02
M0R835	Protein Sf3b6 OS=Rattus norvegicus GN=Sf3b6 PE=1 SV=2 - [M0R835_RAT]	1.691383	1.70E-02
Q4PP99	Cardiac troponin C OS=Rattus norvegicus GN=Tnnc1 PE=1 SV=1 - [Q4PP99_RAT]	0.605755	1.70E-02
Q5RJR8	Leucine-rich repeat-containing protein 59 OS=Rattus norvegicus GN=Lrrc59 PE=1 SV=1 - [LRC59_RAT]	0.606543	1.72E-02
Q3KRE0	ATPase family AAA domain-containing protein 3 OS=Rattus norvegicus GN=Atad3 PE=1 SV=1 - [ATAD3_RAT]	0.606461	1.72E-02
Q63662	UDP-glucuronosyltransferase OS=Rattus norvegicus GN=Ugt1a6 PE=1 SV=1 - [Q63662_RAT]	0.606704	1.73E-02
B2RZD6	Ndufa4 protein OS=Rattus norvegicus GN=Ndufa4 PE=1 SV=1 - [B2RZD6_RAT]	1.687368	1.74E-02
D4A3V2	NADH dehydrogenase [ubiquinone] 1 alpha subcomplex subunit 6 OS=Rattus norvegicus GN=Ndufa6 PE=1 SV=1 - [D4A3V2_RAT]	1.683099	1.78E-02
B1WBV4	Pleckstrin homology domain containing, family F (With FYVE domain) member 2 OS=Rattus norvegicus GN=Plekhf2 PE=1 SV=1 - [B1WBV4_RAT]	1.680272	1.81E-02
P04642	L-lactate dehydrogenase A chain OS=Rattus norvegicus GN=Ldha PE=1 SV=1 - [LDHA_RAT]	1.679612	1.82E-02
D4A5Y7	Protein Rsph9 OS=Rattus norvegicus	0.609796	1.82E-0

	GN=Rsph9 PE=4 SV=1 - [D4A5Y7_RAT]		2
Q675A5	Group XV phospholipase A2 OS=Rattus norvegicus GN=Pla2g15 PE=1 SV=1 - [PAG15_RAT]	0.610236	1.84E-02
Q4TU93	C-type mannose receptor 2 OS=Rattus norvegicus GN=Mrc2 PE=1 SV=1 - [MRC2_RAT]	0.610538	1.85E-02
M0RCH5	Glucosamine-6-phosphate isomerase OS=Rattus norvegicus GN=Gnpda1 PE=1 SV=2 - [M0RCH5_RAT]	1.67268	1.89E-02
Q5PQK2	Fusion, derived from t(1216) malignant liposarcoma (Human) OS=Rattus norvegicus GN=Fus PE=1 SV=1 - [Q5PQK2_RAT]	1.672547	1.89E-02
Q64268	Heparin cofactor 2 OS=Rattus norvegicus GN=Serpind1 PE=1 SV=1 - [HEP2_RAT]	0.612051	1.90E-02
Q9ER34	Aconitate hydratase, mitochondrial OS=Rattus norvegicus GN=Aco2 PE=1 SV=2 - [ACON_RAT]	1.667984	1.94E-02
Q4V8K5	BRO1 domain-containing protein BROX OS=Rattus norvegicus GN=Brox PE=2 SV=1 - [BROX_RAT]	0.613245	1.94E-02
D3ZX63	Protein LOC103692166 OS=Rattus norvegicus GN=Wdr54 PE=4 SV=1 - [D3ZX63_RAT]	0.613651	1.95E-02
G3V9U1	Centaurin, gamma 2 (Predicted) OS=Rattus norvegicus GN=Agap1 PE=4 SV=1 - [G3V9U1_RAT]	0.61374	1.96E-02
P02793	Ferritin light chain 1 OS=Rattus norvegicus GN=Ftl1 PE=1 SV=3 - [FRIL1_RAT]	0.614133	1.97E-02
M0R692	Protein Ighv1-22 OS=Rattus norvegicus GN=Ighv1-22 PE=1 SV=1 - [M0R692_RAT]	1.664085	1.99E-02
D3ZZT9	Protein Col14a1 OS=Rattus norvegicus GN=Col14a1 PE=1 SV=3 - [D3ZZT9_RAT]	1.661589	2.02E-02
F1M7F7	Complement component C6 OS=Rattus norvegicus GN=C6 PE=1 SV=3 - [F1M7F7_RAT]	0.615455	2.02E-02
Q497C3	UPF0585 protein C16orf13 homolog OS=Rattus norvegicus PE=2 SV=2 -	0.615894	2.03E-02

	[CP013_RAT]		
M0R919	Prefoldin subunit 3 OS=Rattus norvegicus GN=Vbp1 PE=1 SV=2 - [M0R919_RAT]	1.659607	2.04E-02
D4A830	Protein Ppa2 OS=Rattus norvegicus GN=Ppa2 PE=1 SV=2 - [D4A830_RAT]	0.61641	2.05E-02
P49432	Pyruvate dehydrogenase E1 component subunit beta, mitochondrial OS=Rattus norvegicus GN=Pdhb PE=1 SV=2 - [ODPB_RAT]	1.657948	2.06E-02
P56574	Isocitrate dehydrogenase [NADP], mitochondrial OS=Rattus norvegicus GN=Idh2 PE=1 SV=2 - [IDHP_RAT]	1.656158	2.08E-02
P04636	Malate dehydrogenase, mitochondrial OS=Rattus norvegicus GN=Mdh2 PE=1 SV=2 - [MDHM_RAT]	1.655139	2.09E-02
F1LP59	Rab3 GTPase-activating protein catalytic subunit OS=Rattus norvegicus GN=Rab3gap1 PE=1 SV=3 - [F1LP59_RAT]	0.617512	2.09E-02
P04904	Glutathione S-transferase alpha-3 OS=Rattus norvegicus GN=Gsta3 PE=1 SV=3 - [GSTA3_RAT]	1.654217	2.10E-02
Q6AZ33	Biliverdin reductase A OS=Rattus norvegicus GN=Blvra PE=1 SV=1 - [Q6AZ33_RAT]	0.618337	2.12E-02
P70623	Fatty acid-binding protein, adipocyte OS=Rattus norvegicus GN=Fabp4 PE=1 SV=3 - [FABP4_RAT]	1.652318	2.13E-02
Q6XDA0	Erythroid spectrin beta OS=Rattus norvegicus GN=Sptb PE=1 SV=1 - [Q6XDA0_RAT]	0.619306	2.15E-02
D3ZLF6	Protein Snrpa1 OS=Rattus norvegicus GN=Snrpa1 PE=1 SV=3 - [D3ZLF6_RAT]	0.619543	2.16E-02
D4A9A3	Protein Cenpv OS=Rattus norvegicus GN=Cenpv PE=1 SV=3 - [D4A9A3_RAT]	1.646259	2.20E-02
M0RA24	Protein LOC103694107 OS=Rattus norvegicus GN=LOC103694107 PE=4 SV=2 - [M0RA24_RAT]	1.642344	2.25E-02
A0A0G2JUF1	Peroxisomal trans-2-enoyl-CoA reductase OS=Rattus norvegicus	1.637786	2.31E-02

	GN=Pecr PE=1 SV=1 - [A0A0G2JUF1_RAT]		
P18437	Non-histone chromosomal protein HMG-17 OS=Rattus norvegicus GN=Hmgn2 PE=1 SV=2 - [HMGN2_RAT]	1.631823	2.39E-02
P62828	GTP-binding nuclear protein Ran OS=Rattus norvegicus GN=Ran PE=1 SV=3 - [RAN_RAT]	1.628968	2.43E-02
A0A096MKH4	Protein Ube2j1 (Fragment) OS=Rattus norvegicus GN=Ube2j1 PE=1 SV=1 - [A0A096MKH4_RAT]	1.626703	2.46E-02
M0R5J4	Uncharacterized protein OS=Rattus norvegicus PE=3 SV=1 - [M0R5J4_RAT]	1.626233	2.47E-02
F1M7X3	Protein Cdh13 OS=Rattus norvegicus GN=Cdh13 PE=1 SV=3 - [F1M7X3_RAT]	1.623711	2.50E-02
P29066	Beta-arrestin-1 OS=Rattus norvegicus GN=Arrb1 PE=1 SV=1 - [ARRB1_RAT]	1.618924	2.57E-02
D3ZUL3	Protein Col6a1 OS=Rattus norvegicus GN=Col6a1 PE=1 SV=1 - [D3ZUL3_RAT]	1.616718	2.61E-02
P16303	Carboxylesterase 1D OS=Rattus norvegicus GN=Ces1d PE=1 SV=2 - [CES1D_RAT]	1.616667	2.61E-02
P08011	Microsomal glutathione S-transferase 1 OS=Rattus norvegicus GN=Mgst1 PE=1 SV=3 - [MGST1_RAT]	1.614325	2.64E-02
A0A0G2JSU4	N-myc downstream regulated gene 2, isoform CRA_b OS=Rattus norvegicus GN=Ndrg2 PE=1 SV=1 - [A0A0G2JSU4_RAT]	1.613054	2.66E-02
P15205	Microtubule-associated protein 1B OS=Rattus norvegicus GN=Map1b PE=1 SV=2 - [MAP1B_RAT]	1.609729	2.71E-02
Q9Z2L0	Voltage-dependent anion-selective channel protein 1 OS=Rattus norvegicus GN=Vdac1 PE=1 SV=4 - [VDAC1_RAT]	1.605601	2.78E-02
D4A3V5	Protein Cgnl1 OS=Rattus norvegicus GN=Cgnl1 PE=1 SV=1 - [D4A3V5_RAT]	1.6	2.87E-02

A0A0G2JSH5	Serum albumin OS=Rattus norvegicus GN=Alb PE=1 SV=1 - [A0A0G2JSH5_RAT]	1.594444	2.96E-02
A0A0G2K2X3	Protein Sf3a3 OS=Rattus norvegicus GN=Sf3a3 PE=1 SV=1 - [A0A0G2K2X3_RAT]	1.58588	3.10E-02
P13803	Electron transfer flavoprotein subunit alpha, mitochondrial OS=Rattus norvegicus GN=Etfa PE=1 SV=4 - [ETFA_RAT]	1.585544	3.11E-02
D3ZRN3	Protein Actbl2 OS=Rattus norvegicus GN=Actbl2 PE=1 SV=1 - [D3ZRN3_RAT]	1.583921	3.14E-02
P41350	Caveolin-1 OS=Rattus norvegicus GN=Cav1 PE=1 SV=3 - [CAV1_RAT]	1.582465	3.16E-02
A0A0G2K484	Protein Myh1 OS=Rattus norvegicus GN=Myh1 PE=1 SV=1 - [A0A0G2K484_RAT]	1.581515	3.18E-02
P69897	Tubulin beta-5 chain OS=Rattus norvegicus GN=Tubb5 PE=1 SV=1 - [TBB5_RAT]	1.577199	3.26E-02
D3ZCQ9	Myeloid leukemia factor 1 (Predicted), isoform CRA_a OS=Rattus norvegicus GN=Mlf1 PE=1 SV=1 - [D3ZCQ9_RAT]	1.571906	3.36E-02
Q561S0	NADH dehydrogenase [ubiquinone] 1 alpha subcomplex subunit 10, mitochondrial OS=Rattus norvegicus GN=Ndufa10 PE=1 SV=1 - [NDUAA_RAT]	1.570492	3.38E-02
O55158	Tetraspanin OS=Rattus norvegicus GN=Tspan8 PE=1 SV=2 - [O55158_RAT]	1.56892	3.41E-02
D3ZFY8	Protein LOC100912618 OS=Rattus norvegicus GN=LOC100912618 PE=1 SV=1 - [D3ZFY8_RAT]	1.568353	3.42E-02
G3V6V1	Aminopeptidase B OS=Rattus norvegicus GN=Rnpep PE=1 SV=1 - [G3V6V1_RAT]	1.566964	3.45E-02
Q03344	ATPase inhibitor, mitochondrial OS=Rattus norvegicus GN=Atpif1 PE=3 SV=2 - [ATIF1_RAT]	1.565445	3.48E-02
D3ZF77	Protein Akr1cl OS=Rattus norvegicus GN=Akr1cl PE=1 SV=1 -	1.562401	3.54E-02

	[D3ZF77_RAT]		
P61589	Transforming protein RhoA OS=Rattus norvegicus GN=Rhoa PE=1 SV=1 - [RHOA_RAT]	1.557439	3.64E-02
P19945	60S acidic ribosomal protein P0 OS=Rattus norvegicus GN=Rplp0 PE=1 SV=2 - [RLA0_RAT]	1.556338	3.66E-02
G3V6H0	Protein LOC100363782 OS=Rattus norvegicus GN=LOC100363782 PE=3 SV=1 - [G3V6H0_RAT]	1.550576	3.78E-02
Q9Z118	Polypyrimidine tract-binding protein 3 OS=Rattus norvegicus GN=Ptbp3 PE=2 SV=1 - [PTBP3_RAT]	1.550797	3.78E-02
Q5XIF6	Tubulin alpha-4A chain OS=Rattus norvegicus GN=Tuba4a PE=1 SV=1 - [TBA4A_RAT]	1.547525	3.85E-02
B5DEP0	Dguok protein OS=Rattus norvegicus GN=Dguok PE=2 SV=1 - [B5DEP0_RAT]	1.546209	3.88E-02
Q63258	Integrin alpha-7 OS=Rattus norvegicus GN=Itga7 PE=1 SV=2 - [ITA7_RAT]	1.543002	3.94E-02
D4A7R3	Nucleoporin 205kDa (Predicted) OS=Rattus norvegicus GN=Nup205 PE=1 SV=1 - [D4A7R3_RAT]	1.539715	4.02E-02
P16228	Cathepsin E OS=Rattus norvegicus GN=Ctse PE=1 SV=3 - [CATE_RAT]	1.536937	4.08E-02
O88656	Actin-related protein 2/3 complex subunit 1B OS=Rattus norvegicus GN=Arpc1b PE=1 SV=3 - [ARC1B_RAT]	1.533708	4.15E-02
B0BNM1	NAD(P)H-hydrate epimerase OS=Rattus norvegicus GN=Apoa1bp PE=2 SV=1 - [NNRE_RAT]	1.533333	4.16E-02
M0R660	Glyceraldehyde-3-phosphate dehydrogenase OS=Rattus norvegicus PE=1 SV=1 - [M0R660_RAT]	1.532961	4.17E-02
B3IYD2	Ufm1-conjugating enzyme 1, isoform CRA_a OS=Rattus norvegicus GN=Ufc1 PE=2 SV=1 - [B3IYD2_RAT]	1.532189	4.19E-02
P45592	Cofilin-1 OS=Rattus norvegicus GN=Cfl1 PE=1 SV=3 - [COF1_RAT]	1.529976	4.24E-02
F7FKI5	Pyruvate dehydrogenase E1 component subunit alpha OS=Rattus norvegicus	1.525708	4.34E-02

	GN=Pdha1 PE=1 SV=2 - [F7FKI5_RAT]		
A0A0G2K2M9	Protein Srrm2 OS=Rattus norvegicus GN=Srrm2 PE=1 SV=1 - [A0A0G2K2M9_RAT]	1.524932	4.36E-02
B2RZC6	Ilf2 protein OS=Rattus norvegicus GN=Ilf2 PE=1 SV=1 - [B2RZC6_RAT]	1.524762	4.36E-02
I6L9G6	Protein Tardbp OS=Rattus norvegicus GN=Tardbp PE=1 SV=1 - [I6L9G6_RAT]	1.52429	4.37E-02
Q6AY18	Protein Ppa1 OS=Rattus norvegicus GN=Sar1a PE=1 SV=1 - [Q6AY18_RAT]	1.521472	4.44E-02
Q6LDP3	Rat glutathione S-transferase OS=Rattus norvegicus GN=Gstm1 PE=2 SV=1 - [Q6LDP3_RAT]	1.521429	4.44E-02
Q99NA5	Isocitrate dehydrogenase [NAD] subunit alpha, mitochondrial OS=Rattus norvegicus GN=Idh3a PE=1 SV=1 - [IDH3A_RAT]	1.521042	4.45E-02
B2RZ77	Dermatopontin (Predicted), isoform CRA_a OS=Rattus norvegicus GN=Dpt PE=1 SV=1 - [B2RZ77_RAT]	1.520087	4.48E-02
Q6P6R2	Dihydrolipoyl dehydrogenase, mitochondrial OS=Rattus norvegicus GN=Dld PE=1 SV=1 - [DLDH_RAT]	1.518868	4.51E-02
